# Extremely Large-Aperture Arrays for V2X Communication, Localization and Sensing

**DOI:** 10.3390/s26051563

**Published:** 2026-03-02

**Authors:** Nicolò Decarli, Caterina Giovannetti, Francesco Guidi, Anna Guerra, Alberto Zanella, Barbara Mavì Masini

**Affiliations:** 1 National Research Council—Institute of Electronics, Computer and Telecommunication Engineering (CNR-IEIIT), 40136 Bologna, Italy; nicolo.decarli@cnr.it (N.D.); alberto.zanella@cnr.it (A.Z.); 2 Department of Electrical, Electronic, and Information Engineering “Guglielmo Marconi” (DEI), University of Bologna, 40126 Bologna, Italy; anna.guerra3@unibo.it (A.G.); 3 National Laboratory of Wireless Communications of National Inter-University Consortium for Telecommunications, (WiLab—CNIT), 40133 Bologna, Italy

**Keywords:** V2X, integrated sensing and communication (ISAC), near-field, extremely large-aperture array (ELAA), extra-large multiple-input multiple-output (XL-MIMO), localization

## Abstract

Future sixth-generation (6G) vehicular networks are expected to support connected, autonomous, and cooperative driving through ultra-reliable, low-latency, and context-aware vehicle-to-everything (V2X) communications. In this paper, we investigate the role of extremely large-aperture arrays (ELAAs) deployed on board vehicles as a key enabler for the joint enhancement of communication, localization, and sensing functionalities, with particular focus on V2X sidelink transmissions. Leveraging the large spatial aperture of ELAAs, advanced beamfocusing and line-of-sight (LOS)-multiple-input multiple-output (MIMO) techniques are reviewed to improve communication reliability and spatial multiplexing in highly dynamic vehicular scenarios. Moreover, we analyze vehicular near-field single-anchor localization and sensing enabled by large arrays, as well as predictive beamforming strategies based on Doppler analysis that enable the estimation of multiple velocity components of surrounding objects and vehicles. This paper highlights the tight interplay between communication and sensing, demonstrating how it can enhance performance across these domains. Our analysis provides insights into the potential of ELAA-based integrated sensing and communication (ISAC) architectures for next-generation vehicular networks.

## 1. Introduction

The evolution toward sixth-generation (6G) vehicular networks is driven by the stringent requirements of connected, autonomous, and cooperative driving, which demand ultra-reliable, low-latency, and highly accurate communication, localization, and sensing capabilities [1]. Vehicle-to-everything (V2X) communications, and in particular sidelink transmissions, play a central role in enabling direct information exchange between vehicles without relying on network infrastructure [2]. In such highly dynamic and frequently interference-limited environments, conventional far-field communication and positioning paradigms may be insufficient to meet the performance requirements of advanced vehicular applications.

Recent advances in antenna integration and radio-frequency front-end design, together with the availability of large physical surfaces on board vehicles, could enable the deployment of extremely large-aperture arrays (ELAAs) for V2X applications. Unlike traditional arrays, ELAAs exhibit a physically large dimension, which results in an aperture spanning a large number of wavelengths. In this manner, they naturally operate in the radiative near-field region for typical inter-vehicular distances, especially at high carrier frequencies [3]. This propagation regime provides enhanced spatial resolution and introduces new degrees of freedom (DoFs) that can be exploited to jointly improve communication, localization, and sensing. As a result, near-field signal processing emerges as a potential key technological enabler for next-generation vehicular networks.

The distinction between far-field and near-field regimes has significant implications for both communication and sensing. In the far field, electromagnetic waves can be approximated as planar, and propagation is mainly characterized by angular information, enabling conventional beamforming. In contrast, in the near field, the wavefront is curved (spherical, or often approximated as parabolic), and the received signal depends jointly on angle and distance. In this condition, the classical beamsteering concept can be generalized to beamfocusing, i.e., concentrating electromagnetic energy within a finite spatial region rather than along an angular direction [4]. This capability is particularly appealing for V2X sidelink, where multiple vehicles can be approximately aligned in angle (e.g., along the same lane) but separated in range: range-dependent spatial selectivity can improve interference control, spatial reuse, and robustness in dense traffic. Moreover, when both transmitter and receiver are equipped with multi-antenna arrays, near-field propagation can support line-of-sight (LOS) spatial multiplexing with multiple communication modes, enabling very high-data-rate short-range links between nearby vehicles even in limited-scattering environments [5].

Beyond communications, extra-large apertures can act as high-resolution spatial sensors. The curvature of the received wavefront across an ELAA embeds joint angle and range information, enabling single-anchor relative localization without relying on multiple infrastructure anchors and without necessarily requiring wideband ranging or tight time synchronization [6]. Moreover, the physical extension of ELAAs enhances Doppler processing and can enable the precise estimation of relative motion, opening the door to predictive beamforming and proactive link maintenance in highly dynamic vehicular scenarios [7]. These features naturally fit within the broader vision of integrated sensing and communication (ISAC), where a common radio platform jointly supports connectivity and environment awareness [8].

Motivated by these considerations, this paper investigates ELAA-enabled near-field techniques for V2X sidelink, with the goal of highlighting how extra-large vehicular apertures can jointly enhance communications, localization, and sensing.

### 1.1. State of the Art

While new radio (NR)-V2X sidelink standardization is mature and evolving [9,10] and ISAC is rapidly expanding as a 6G pillar [11], the intersection of vehicular ISAC with near-field/ELAA operation remains mostly under-explored. This gap motivates system and signal models that explicitly capture near-field propagation and extra-large apertures in V2X settings and that quantify the resulting implications for joint communication and sensing performance under realistic vehicular constraints.

Vehicular connectivity has evolved from IEEE 802.11p/ITS-G5 and long-term evolution (LTE)-based cellular-V2X (C-V2X) toward fifth-generation (5G) NR-V2X solutions that can jointly support safety-critical messaging and data-intensive cooperative perception. Comprehensive overviews of the NR sidelink design and its evolution beyond Rel-16 highlight key enablers such as flexible numerologies, improved reliability/latency mechanisms, and enhanced resource allocation procedures for advanced V2X services [2,9,10,12]. In parallel, 3GPP releases after Rel-16 (including 5G-Advanced starting from Release 18) continue to refine sidelink operation and related features to accommodate higher automation levels and new use cases [13,14].

To sustain the growing throughput demands of cooperative perception (e.g., raw or compressed sensor sharing), millimeter-wave (mmWave) V2X communications have attracted major interest thanks to the availability of large bandwidths and the possibility to pack a large number of antennas into a small area [15]. MmWave vehicular communications represent a fundamental technology for enhancing V2X connectivity, enabling advanced applications such as autonomous driving, road safety, and intelligent traffic management. The use of mmWave frequencies offers significantly larger bandwidths, enabling ultra-high-speed data transmission and low-latency communication, both critical for real-time vehicular interactions. As research continues, innovations in antenna design, adaptive communication protocols, and artificial intelligence (AI)-driven network optimization will further unlock the full potential of mmWave for V2X applications [15,16]. However, despite these advantages, mmWave communications also face significant challenges [17,18]. Higher-frequency signals experience greater attenuation, limiting their effective range compared to lower-frequency signals. Additionally, mmWave signals are highly susceptible to obstacles, such as buildings and vehicles, as well as adverse weather conditions, including rain and snow, which can disrupt transmission reliability [19]. To mitigate these limitations, modern V2X communication systems are expected to employ advanced techniques such as beamforming, which focuses signal energy in specific directions. Furthermore, integrating mmWave with sub-6 GHz frequencies and leveraging emerging 5G and future 6G networks can enhance both the robustness and coverage of V2X communication [19,20].

In addition to communications, modern vehicles rely heavily on radar sensing for object detection and tracking. A large number of automotive radar deployments operate in the 77–81 GHz band, which has been harmonized in Europe for automotive short-range radars through ECC decisions and ETSI standards [21,22]. The co-location of high-frequency vehicular communications and radar sensing in the adjacent (or potentially shared) spectrum motivates tighter integration between the two functionalities, both to improve resource efficiency and to enable new cooperative perception capabilities. Therefore, ISAC has emerged as a key research direction for beyond-5G/6G systems, aiming to reuse waveform, hardware, spectrum, and signal processing to serve both data transfer and environmental awareness [11,23]. For vehicular networks, ISAC is particularly promising, as it allows vehicles to communicate with each other while concurrently sensing their surroundings, including the positions, velocities, and types of other vehicles and obstacles. This dual-use capability is expected to provide a more holistic understanding of the environment, supporting more informed decision-making for autonomous systems [24,25,26]. The literature spans waveform and precoder design, receiver processing, sensing–communication trade-offs, and network-level coordination. Yet, many ISAC formulations still adopt simplifying assumptions (e.g., far-field plane-wave propagation and relatively compact arrays) that may not hold for vehicular platforms equipped with extra-large apertures and distributed antennas.

Indeed, the push toward ELAAs and, in general, extremely large-scale MIMO (XL-MIMO) makes near-field effects increasingly relevant: the wavefront becomes spherical, and angle and range become jointly encoded in the spatial signature. XL-MIMO can use tens to hundreds of antennas to focus energy into small spatial regions, greatly increasing spectral and energy efficiency and robustness to interference [27]. As arrays become extremely large in aperture, spatial non-stationarities may arise: different parts of the array see different clusters or even different users, and each user only illuminates a visibility region over part of the array [28,29]. This XL-MIMO regime changes channel modeling, beamforming, and user scheduling but enables many terminals to be served with high rates in crowded scenarios [30]. In such large arrays, near-field conditions dominate; beamforming must depend on user position, not just angle, and non-stationarity can be exploited to reduce transceiver complexity via subarray architectures and visual-region-aware scheduling. These XL-MIMO concepts are beginning to be linked to 6G-V2X, where very long roadside or building-integrated arrays could serve as dense traffic corridors. Unlike traditional antenna arrays, ELAAs naturally operate in the radiative near-field region for typical inter-vehicular distances, especially at high carrier frequencies. Tutorials and surveys on XL-MIMO and near-field communications emphasize that this regime can unlock additional spatial DoFs and improved localization/sensing resolution, but it also introduces new channel models and signal processing strategies [31,32].

Most V2X antenna work today implements moderate-sized arrays on vehicles or infrastructure. In [33], the authors consider a sub-6 GHz and mmWave 8-element MIMO array for 5G NR-V2X and use four horizontally arranged and four orthogonal vertical tapered slot elements to realize $360^{\circ }$ azimuth coverage and high gain, highlighting that conventional phased/lens arrays become complex and lossy when scaled to large sizes. A compact wide-angle scanning multibeam array at 5.9 GHz with closely spaced elements with a 3 dB scanning range of $\pm 120^{\circ }$ is considered in [34] while mitigating mutual coupling via slots and decoupling structures with a design intended to be scalable to mmWave frequencies. A reconfigurable mmWave planar phased array providing vertical beam tilting up to $\pm 40^{\circ }$ and azimuth beam scanning ($\pm 32^{\circ }$) in the N257 band, with >8 dBi gain and >20 dB isolation, mounted on a car model for 5G NR-V2X demonstrates practical large-aperture behavior (hybrid structure and wide spatial coverage) in vehicular form factors [35]. Designs integrating mmWave active phased arrays with wideband sub-6 GHz antennas show that dual-band systems can be built with high isolation and large mmWave scan ranges [36]. This points toward densely integrated hybrid arrays on vehicles. A V2X sidelink localization framework explicitly leveraging near-field propagation from large arrays to enable accurate vehicle-to-vehicle (V2V) positioning without extra anchors or tight synchronization was proposed in [37].

Despite rich work on massive XL-MIMO theory, explicit V2X implementations with truly extremely large arrays (e.g., hundreds/thousands of elements) are still scarce. Most vehicular designs focus on few-element arrays or small multi-beam panels [33,34,35,38], indicating local coverage (per-vehicle or per-RSU), rather than continuous, XL-aperture roadside deployments, which are tailored for communication performance; joint designs for communication, localization, and sensing with XL arrays are only beginning to appear.

### 1.2. Contribution

This paper discusses how near-field techniques enabled by vehicular ELAAs can bring substantial benefits across communication, localization, and sensing functionalities in 6G-V2X systems. Particular attention is devoted to sidelink-based V2X scenarios, which are especially relevant for safety-critical applications such as cooperative driving and platooning.

From a communication standpoint, the large spatial aperture of ELAAs enables near-field beamfocusing, which can be exploited in multi-user MIMO configurations to improve interference robustness and spatial reuse. By concentrating energy on specific spatial regions rather than angular directions, near-field beamfocusing allows more precise separation of users located at similar angles but different distances. In addition, we analyze communication between two vehicles equipped with multiple antennas operating in near-field LOS conditions. In this case, LOS-MIMO transmission can be leveraged to increase the channel rank (i.e., the achievable DoF), with a strong dependence on the array dimensions, geometry, and inter-vehicular distance. This analysis provides insights into how near-field propagation can be exploited to enhance channel capacity in short-range V2X links.

Beyond communication, vehicular ELAAs enable accurate localization and sensing capabilities even in the absence of network-side anchors. In this paper, we discuss the feasibility of single-anchor localization using an ELAA-equipped vehicle. Two complementary approaches are considered. First, we analyze classical localization techniques, where a transmitting vehicle is localized by an ELAA receiver using near-field spatial signatures. Second, we consider radar-based localization and sensing, where the position and kinematic parameters of another vehicle are inferred from reflected signals. These approaches are particularly effective at short inter-vehicular distances, such as in platooning scenarios, where near-field effects are dominant and high spatial resolution is required.

Furthermore, we analyze predictive beamforming strategies enabled by Doppler analysis on large antenna arrays. Thanks to the spatial resolution of ELAAs, Doppler processing can be used to estimate multiple components of the relative velocity vector, thereby allowing prediction of future beam directions and focal points. This concept is analyzed for both active communication scenarios involving direct transmission between two vehicles and sensing-based scenarios that rely on reflected signals. Predictive beamforming is shown to be a key enabler for maintaining reliable links and sensing accuracy in highly dynamic vehicular environments.

The manuscript is organized following a tutorial-oriented structure. It first introduces the fundamental characteristics of vehicular scenarios, with a particular emphasis on communication technologies and on representative use cases in which communication, localization, and sensing functionalities may be jointly exploited, especially in relation to near-field operations, which constitute the core contribution of this work. As a general methodology, for each application specifically contextualized to the vehicular domain, the relevant signal and system models are presented, together with the most appropriate performance metrics. Dedicated numerical examples are provided to quantitatively illustrate both the potential benefits and the inherent limitations of applying the considered techniques in vehicular environments. The main challenges and critical aspects, partially those discussed within each application-specific subsection, are subsequently consolidated and examined in greater depth in the section devoted to future research directions.

The remainder of this paper is organized as follows: Section 2 discusses V2X communications and representative use cases that particularly motivate high-rate selective links and localization-aware operation. Section 3 reviews preliminaries on large arrays and near-field propagation. Section 4 and Section 5 analyze ELAA-enabled near-field communication, localization, and sensing, including beamfocusing, near-field LOS-MIMO, single-anchor localization, and Doppler-based predictive beamforming. Throughout this paper, numerical examples are provided to illustrate the impact of array size; carrier frequency, covering frequency ranges from FR1 to FR2 and FR3; and inter-vehicular distance on communication, localization, and sensing performance. Finally, Section 6 concludes this paper by summarizing the key insights and outlining open challenges and promising research directions for ELAA-enabled near-field ISAC in future 6G vehicular networks.

## 2. V2X Communication and Use Cases

This section first reviews the main features of 5G-V2X connectivity from a standardization perspective, with a particular emphasis on sidelink communications and their evolution toward 6G systems. Subsequently, the principal use cases are analyzed, discussing the interplay between communication and localization/sensing in vehicular environments, as well as the key challenges characterizing such operational scenarios. This discussion highlights the most relevant implications for near-field operations, which are addressed in the subsequent sections.

### 2.1. 5G-V2X and Beyond

V2X connectivity is a key enabler for connected and cooperative driving, as it allows vehicles to exchange safety-critical messages and, increasingly, rich sensor-derived information with nearby road users and roadside infrastructure. Current deployments and standardization efforts are characterized by the coexistence of two main families of solutions: IEEE 802.11p-based approaches (e.g., ETSI ITS-G5) and C-V2X solutions standardized by 3GPP. In Europe, both families are considered within the 5.9 GHz band, whose harmonized use is regulated at the CEPT/ECC level and supported by ETSI standards for ITS radio equipment [39,40]. Within the 3GPP ecosystem, V2X services can be delivered over both the conventional cellular interface (Uu) and the direct device-to-device interface (PC5, a.k.a. sidelink). The system architecture explicitly supports both modes and enables service continuity and interworking between LTE and NR radio access, allowing operators and manufacturers to mix network-assisted and infrastructure-less operations depending on coverage and service requirements [41]. From a service perspective, 3GPP specifies V2X requirements spanning safety (e.g., cooperative awareness and collision avoidance), advanced driving (e.g., platooning), and non-safety applications (e.g., infotainment and sensor sharing), with stringent latency and reliability targets for the most demanding cases [42,43]. The C-V2X roadmap has evolved from LTE-V2X (introduced in Releases 14/15) to 5G NR-V2X (introduced in Releases 16), with the latter designed to better support advanced V2X services through a more flexible physical layer and improved resource allocation mechanisms. In 5G NR-V2X, sidelink operation is extended beyond broadcast to also support unicast and groupcast while leveraging NR features such as scalable numerologies and more advanced link adaptation options [10,44,45]. These capabilities are particularly relevant for scenarios in which vehicles need reliable low-latency exchanges with selected neighbors (e.g., within a platoon) or need to sustain high-rate short-range links for sharing rich perception information. Beyond Release 16, 3GPP continues to enhance sidelink as part of the broader 5G-Advanced evolution. Release 17 includes sidelink enhancements among its highlights, reflecting the standardization focus on improving reliability, coverage, and efficiency, as well as enabling new sidelink features beyond the original V2X scope [46,47,48]. Release 18, as the first 5G-Advanced release, further extends the sidelink feature set, with the objective of increasing sidelink throughput and supporting broader operating conditions (including higher-frequency operation and improved sidelink evolution mechanisms) [14,49,50]. While the exact feature maturity and deployment timelines depend on chipset and ecosystem readiness, this roadmap confirms sidelink as a long-term pillar for direct V2X connectivity.

Looking ahead, beyond-5G and -6G research trends point toward three directions that are particularly impactful for V2X: multi-band operation combining sub-6 GHz coverage layers with wideband high-frequency links (mmWave and potentially new mid-bands), pervasive use of large and distributed antenna arrays to provide high beamforming gain and spatial selectivity, and tighter integration of communications with localization and sensing (i.e., ISAC). These trends align with the broader IMT-2030 framework, which highlights new capabilities and use cases for the 2030+ timeframe [51].

### 2.2. Use Cases

V2X communication, localization and sensing have to be designed for a wide range of operating contexts, from sparse high-speed roads with long communication ranges and low interference to dense highways with strong Doppler and mutual occlusions, complex 3D interchanges dominated by non-line-of-sight (NLOS) conditions and complicate geometry, and urban cores where multipath, vulnerable road-user (VRU) density, and global navigation satellite system (GNSS) degradation factors are severe; additional challenges arise in rural villages and mountain roads with winding, sloped routes; intermittent coverage; and frequent NLOS. In these environments, the set of traffic participants is highly diverse (cars, trucks, buses and light rail, motorcycles and micromobility devices, VRUs, and potentially drones) leading to large variability in kinematics, radar signatures, and communication capabilities and motivating multi-modal, role-adaptive designs. Moreover, communication, localization, and sensing must remain effective in infrastructure-denied situations such as tunnels, underpasses, and underground parking (GNSS outages/biases) and under variable cellular quality of service where sidelink may be the only viable option. Finally, because adoption will be gradual and connected and autonomous vehicles will coexist with legacy non-connected traffic, there will be the need to jointly support cooperative, device-based localization and device-free sensing of non-cooperative subjects, without over-relying on universal connectivity. A set of V2X use cases that particularly motivate the technical directions addressed in this paper is reported in the following section.

**Collective perception and extended sensors:** Connected automated vehicles increasingly benefit from collective perception, in which multiple agents share information about the objects and regions they perceive, thereby extending sensing beyond local LOS and mitigating occlusions (e.g., hidden pedestrians, vehicles behind trucks, or objects around corners). ETSI specifies the Collective Perception Service (CPS) and the corresponding collective perception message (CPM), which can include the transmitter’s sensor capabilities, perceived objects, and perception regions. This type of information exchange naturally moves beyond the cooperative awareness message (CAM) and decentralized environmental notification message (DENM) paradigm: it is more data-intensive, must be refreshed frequently to remain useful, and is often most relevant to a subset of nearby vehicles (e.g., those approaching the same intersection, merging area, or conflict zone). A further step is high-definition sensor sharing, where vehicles exchange richer representations (e.g., dense object lists, feature-level representations, or selected sensor streams) to support automated driving functions. This creates a demand for short-range, high-capacity V2V links with stringent latency and reliability, typically between specific neighbor vehicles and/or between vehicles and infrastructure elements that act as perception hubs.

**Cooperative driving and maneuver coordination.** Cooperative maneuvers such as lane changes, lane merges, intersection coordination, and coordinated braking require fast and reliable exchange of intentions and maneuver-related states among the participants. Unlike baseline awareness, these interactions are inherently group-related: only the vehicles that are directly involved (or that may become involved within a short time horizon) need to exchange the most detailed information. In 3GPP groups, many of these scenarios are under enhanced V2X service categories (e.g., advanced driving), where the system must support low-latency and highly reliable connectivity to enable safe trajectory coordination.

Platooning represents an extreme case of cooperative driving in which vehicles travel at short headways and must maintain stable formation under dynamic conditions. While broadcast awareness remains useful, stable platoon control and rapid reaction to disturbances benefit from highly reliable, low-latency exchanges within a well-defined group and may be potentially complemented by higher-rate sharing of local perception or control-relevant information.

**Remote driving and teleoperation support.** Remote driving and teleoperation scenarios introduce a different but equally demanding traffic pattern: uplink transmission of high-quality sensor data (typically video and/or fused perception outputs) from the vehicle to a remote operator or control function, combined with downlink command and feedback streams. Even when remote driving is used as a fallback (e.g., in edge cases for automated driving), it requires robust end-to-end performance and can generate substantial data volumes. In addition, V2X links may be used to relay information or to enhance situational awareness around the teleoperated vehicle.

**Localization-critical interactions and the role of sensing integration.** Many of the above use cases are not only communication-limited but also localization-limited. Cooperative maneuvers and collective perception require accurate relative position, heading, and timing consistency between participants; otherwise, shared object lists and intentions cannot be safely fused or acted upon. While the GNSS can provide a global reference, its availability and integrity may degrade in urban canyons, tunnels, or adverse conditions, motivating complementary localization mechanisms. In this context, high-accuracy positioning requirements in 5G systems and beyond have been widely recognized, and they align with the broader trend of integrating localization capabilities into the communication system. These considerations motivate ISAC approaches, where the same radio hardware and signals contribute to both data exchange and environment state estimation. For short-range vehicular interactions, the ability to support joint communication and sensing/localization becomes particularly attractive because it can reduce overhead, improve robustness, and enable more consistent real-time perception sharing.

#### Implications for ELAA Near-Field-Enabled V2X

In the vehicular context, the availability of large physical surfaces on vehicles makes ELAAs a realistic architectural option, especially at higher carrier frequencies. The presented use cases, in particular collective perception, advanced driving/platooning, and remote driving, motivate the technical requirements that are central to this paper:

- High-data-rate short-range links: Richer perception information (beyond CAM/DENM) can require wideband transmissions and high spectral efficiency, especially under mobility.
- Selective connectivity among specific neighbors: Many interactions are pairwise or group-specific, favoring unicast/groupcast links and spatial selectivity rather than pure broadcast.
- Accurate relative localization and sensing support: Reliable fusion of shared perception and safe maneuver coordination require precise relative geometry estimation, ideally integrated with the communication process.

These requirements provide a clear rationale for investigating ELAAs on vehicles and the resulting near-field regime, where spatial focusing and high-resolution parameter estimation can jointly benefit communications and sensing/localization in V2X sidelink scenarios, thus motivating the analysis developed in the remainder of this paper.

To make the link between the above use cases and the technical analysis more explicit, we mainly refer to three representative operating contexts: (i) multi-lane highways and platoons, where several vehicles are quasi-aligned in angle but separated in range; (ii) dense urban intersections, where rapid geometry changes and NLOS/partial blockage challenge both communication and localization; and (iii) cooperative V2V/V2I perception clusters, where a subset of nearby agents exchange high-rate sensor-derived data and require consistent relative positioning.

## 3. Preliminaries: Large Antenna Arrays and Near-Field Operations

Consider an antenna array with physical aperture *D*. As an illustrative example, the array can be implemented as a uniform linear array (ULA) composed of *N* antenna elements with inter-element spacing δ, yielding an overall aperture D≈Nδ.

A point located at a distance *r* from the array is said to lie in the *far-field region* of the array when its distance exceeds the *Fraunhofer distance* $r_{\mathrm{ff}}$, which is conventionally defined as [52]

(1) $$r_{\mathrm{ff}}=\frac{2D^{2}}{\lambda },$$

where $\lambda =\frac{c}{f_{c}}$ denotes the wavelength, *c* is the speed of light, and $f_{c}$ is the carrier frequency. Conversely, when $d<r_{\mathrm{ff}}$, the point lies in the *near-field region*, also referred to as the *Fresnel region* (we do not consider operations in the *reactive near-field region* in the proximity of the array). Accordingly, for a transmitting array, a receiver is said to operate in the near field if its distance from the transmitting array is smaller than $r_{\mathrm{ff}}$. Similarly, for a receiving array, near-field operation occurs when the transmitter is located within the Fraunhofer distance of the receiving array. In radar systems, the radar is said to operate in the near field when the target lies within $r_{\mathrm{ff}}$ with respect to the transmitting and/or receiving array. From (1), it follows that a near-field operation becomes more likely as the array aperture increases (a geometry-related aspect) and/or the wavelength decreases (a signal-related aspect), which is typical of large-scale arrays operating at high carrier frequencies.

In the near-field region, the plane-wave approximation is no longer valid [27]. As a result, signal modeling must explicitly account for the distance between each individual antenna element of the array and the transmitter/receiver/target, depending on the system under consideration. In particular, the beamforming (or steering) vector becomes a function of both angle and range, in contrast to far-field models, where it depends solely on the angle of departure or arrival [27].

It is important to emphasize that the Fraunhofer distance in (1) is a conventional definition, derived by imposing a maximum tolerable phase error with respect to the plane-wave approximation [53]. In practice, the transition between near-field and far-field regimes is not abrupt; rather, near-field effects vary smoothly with distance. Moreover, such effects are most pronounced along the array’s boresight direction (i.e., the broadside direction, for which the Fraunhofer distance is defined) and gradually diminish as the observation angle increases, eventually becoming negligible in the end-fire direction of the array [54,55].

In the following section, we consider vehicular communications and analyze how the Fraunhofer distance may vary when assuming that the entire front of a vehicle (car or truck) is fitted with an ELAA. We consider three representative array apertures, namely 1.5 m, 2 m, and 2.5 m, which correspond approximately to the front of a small car, a large car, and a truck, respectively. Table 1 reports the corresponding Fraunhofer distances $r_{\mathrm{ff}}=2D^{2}/\lambda$ for four different carrier frequencies used or foreseen for future vehicular and ISAC scenarios: 5.9 GHz (i.e., FR1), 10 GHz (i.e., FR3), 28 GHz, and 60 GHz (i.e., FR2).

The Fraunhofer distance increases quadratically with the array aperture and inversely with the wavelength. For a small car with a 1.5 m aperture array, the near-field region extends to about 89 m at 5.9 GHz and 900 m at 60 GHz. For a truck-sized array of 2.5 m, these distances increase substantially, reaching more than 1 km even at 28 GHz and 2.5 km at 60 GHz. These results indicate that, in practical vehicular scenarios, large-aperture arrays operating at high frequencies can easily generate significant near-field regions. Consequently, near-field effects must be carefully considered when designing communication and sensing operations, as the plane-wave approximation may not hold for typical vehicle-to-vehicle or vehicle-to-infrastructure distances.

It is important to note that, as repeatedly shown in the literature, the Fraunhofer distance is typically much larger than the regions in which near-field phenomena can be effectively exploited to enhance communication and sensing. A more realistic estimate of a *useful* distance for such purposes is roughly one tenth of the Fraunhofer distance [53,56]. Nonetheless, by considering the possibility of ELAAs and high carrier frequencies that lead to the values in Table 1, even one tenth of the Fraunhofer distance can result in significant near-field effects at practical operational ranges.

Finally, it is necessary to emphasize that covering such large apertures with half-wavelength-spaced arrays, as commonly assumed, could be extremely complex if traditional schemes in terms on RF chains and signal processing are adopted. The results reported in Table 2 clearly highlight the strong dependence of the required number of antenna elements on the operating frequency for a fixed physical aperture. While sub-10 GHz operation allows meter-scale apertures to be covered with a relatively limited number of elements, the element count increases dramatically as the carrier frequency moves toward the mmWave regime. For instance, increasing the frequency from 5.9 GHz to 60 GHz results in an order-of-magnitude growth in the number of λ/2-spaced elements for the same aperture, directly translating into higher hardware costs, increased power consumption, and substantially more complex RF front-end and processing architectures. This scaling issue becomes even more critical for planar arrays, where the total number of elements grows quadratically, quickly reaching impractical levels for large apertures. These considerations pose significant challenges for the deployment of large-scale arrays at high frequencies. To mitigate such complexity while preserving a large effective aperture, several alternative solutions can be envisioned, including sparse arrays, sub-array-based architectures, hybrid analog–digital beamforming, and dynamically reconfigurable or holographic surfaces [57,58,59,60,61]. Additionally, operating in intermediate frequency ranges (e.g., FR3) could offer a favorable trade-off between aperture size and system complexity, making them particularly attractive for practical large-aperture ISAC deployments.

In the rest of the manuscript, a V2V scenario is considered for communication- and localization-related applications. However, different interactions can also be realized between moving vehicles and fixed nodes, e.g., roadside units (RSUs), if the array size on board a vehicle or at the RSU, the relative distance, and the operating frequency satisfy the near-field conditions discussed here.

### Signal Model

In the following section, orthogonal frequency-division multiplexing (OFDM) transmission is considered, as it represents the standard for 5G and is also foreseen as an enabler for 6G applications. Considering multiple transmitting antennas (specific cases depending on the different communication/localization applications will be detailed in each of the following sections), the signal emitted by the *n*-th antenna, in equivalent low-pass, is given by

(2) $$s_{n}\left(t\right)=\sum_{m=0}^{M-1}\left(\sum_{k=0}^{K-1}x_{n}\left[k,m\right]e^{j2\pi \frac{k}{T}t}\right)g\left(t-mT_{s}\right)\;,$$

where $x_{n}\left[k,m\right]$ denotes the transmitted data at the kth subcarrier of the mth OFDM symbol at the nth transmit antenna, with *M* OFDM symbols and *K* subcarriers. The function g(t) represents the pulse shaping filter. Δf=1/T is the subcarrier spacing, and $T_{s}=T+T_{\mathrm{CP}}$ corresponds to the duration of one OFDM symbol, including the cyclic prefix (CP) of length $T_{\mathrm{CP}}$.

## 4. ELAA for V2X Communications

This section investigates how near-field techniques can be exploited in the context of vehicular communications. In particular, the potential of near-field operations enabled by large-aperture antenna arrays to enhance spatial multiplexing and multi-user capabilities in vehicular scenarios is analyzed.

In Section 4.1, beamfocusing is considered to enable multi-user MIMO communications between a transmitting vehicle equipped with an ELAA and multiple receiving vehicles, each equipped with a single-antenna terminal. By leveraging near-field beamfocusing, the transmitter can spatially discriminate users using both the angular and distance dimensions, thereby enabling simultaneous transmissions to multiple vehicles.

In Section 4.2, the analysis is extended to the case where both transmitting and receiving vehicles are equipped with multiple antennas. This configuration enables multiple communication layers by exploiting near-field propagation properties, even under LOS conditions. The use of near-field spatial DoFs enables enhanced multiplexing performance beyond what is achievable with conventional far-field MIMO techniques.

### 4.1. Beamfocusing

**Figure 1:**
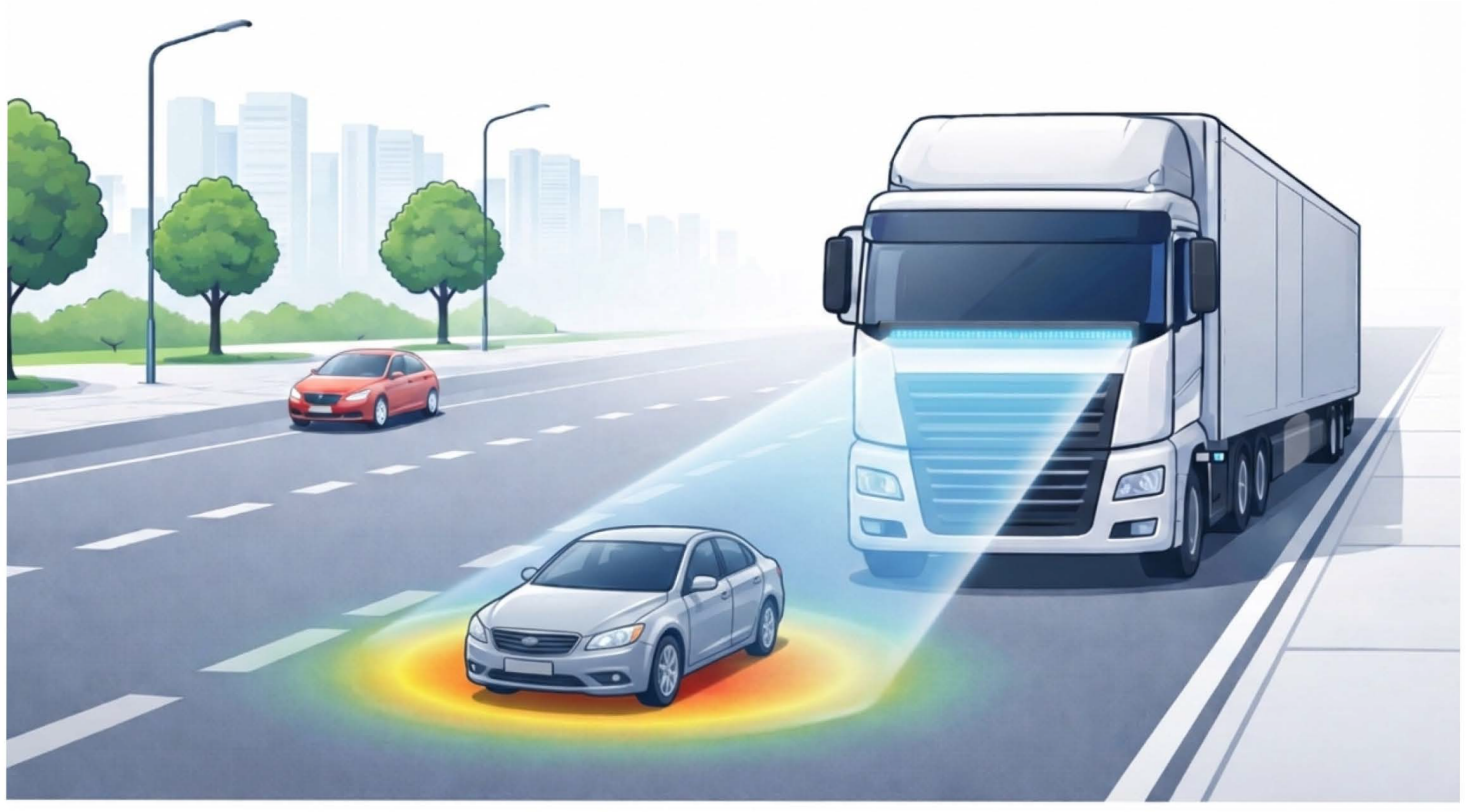
Pictorial example of beamfocusing applied to the V2X scenario, where an ELAA on board the truck realizes a focused spot at the receiving vehicle.

In conventional far-field beamforming, the energy transmitted by an antenna array is primarily steered toward a desired angular direction. Under this condition, the constructive interference of the signals emitted from the individual elements of the transmitting array extends along the angular direction, resulting in a beam that maintains its shape over long distances. However, when the receiver lies within the Fresnel region of an ELAA (where the plane-wave approximation no longer holds and the wavefront must be modeled as spherical), the superposition of the signals from the array elements does not produce an infinitely extended beam, but rather a spatially localized focal region, often referred to as a “spot.” Within this spot, the path lengths from each array element to the receiver align such that the transmitted signals add coherently in phase, yielding a high-intensity focus. Outside this region, destructive interference rapidly reduces the signal power, naturally confining the energy in a finite volume of space. This phenomenon, applied to a vehicular scenario, is pictorially described in Figure 1, where the presence of the focal spot is highlighted. In particular, the truck equipped with the ELAA focuses the beam on the car in front of it, exploiting near-field propagation.

Specifically, consider a transmitting vehicle equipped with the ELAA. The transmit signal vector across antennas, with elements $x_{n}\left[k,m\right]$, for k=0,…,K−1 and m=0,…,M−1, is given by $x\left[k,m\right]=ws\left[k,m\right]\in C^{N\times 1}$, where $w\in C^{N\times 1}$ is the precoding vector and s[k,m] is the modulation symbol. When realizing beamfocusing, the near-field precoding vector is designed such that it compensates for the channel phase at a specific location $p={\left[\theta ,r\right]}^{T}$ where the receiver is supposed to be located. Specifically, $w\left(\theta ,r\right)\in C^{N\times 1}$ is given by

(3) $$\begin{array}{c} w\left(\theta ,r\right)=\left[\begin{array}{c} e^{j\frac{2\pi f_{c}}{c}\left(r_{1}-r\right)}\\ \vdots \\ e^{j\frac{2\pi f_{c}}{c}\left(r_{n}-r\right)}\\ \vdots \\ e^{j\frac{2\pi f_{c}}{c}\left(r_{N}-r\right)} \end{array}\right], \end{array}$$

where $r_{n}$ denotes the distance between the *n*-th transmitting antenna and the position p at distance *r* with respect to the array center.

This spatial confinement has profound implications for multi-user MIMO in vehicular communications. In particular, a transmitting vehicle equipped with an ELAA could simultaneously serve multiple receivers located at similar angles but different distances. By exploiting both the angular and radial dimensions, near-field beamfocusing permits the transmitter to spatially discriminate users that would be indistinguishable in the far-field regime. This reduces inter-user interference and enhances link reliability, even in dense LOS vehicular scenarios such as platooning or urban corridors. On multi-lane highways and platoons, beamfocusing is especially relevant when several neighbors share similar angles of arrival (AoAs) (same lane direction) but differ in range; range-aware selectivity enables spatial reuse (e.g., parallel unicast/groupcast) while suppressing interference toward unintended vehicles. In fact, the ability to focus energy into distinct spots can improve communication efficiency by concentrating power where it is needed and minimizing wasting energy in other locations and unintended exposure to other vehicles or users. Thus, near-field beamfocusing transforms the classical MIMO design paradigm by introducing range-dependent spatial selectivity [4].

The above benefits come with non-negligible practical challenges that are primarily related to channel state information (CSI) acquisition in the near field. In far-field beamforming, it is often sufficient to estimate (or scan) the dominant angular direction, whereas near-field beamfocusing requires knowledge of both angle and range, since the array response depends on the spherical wavefront geometry. As a consequence, the beam training and channel estimation problem becomes intrinsically two-dimensional (polar domain) [62,63], typically requiring denser codebooks and a larger training overhead compared to classical angle-only beamsteering [56,64]. This issue is more pronounced for vehicular applications due to fast formation variations, which demand frequent updates to the focusing point to avoid a rapid loss of beamforming gain, especially when the focal spot exhibits a small beam depth, i.e., when vehicles are at a short distance, as will be analyzed in the following numerical example. Importantly, the same near-field structure that complicates beam training can be exploited for localization. Since the phase curvature across a large aperture embeds joint range and angle information, pilots used for channel acquisition in communication can simultaneously support single-anchor relative positioning, as discussed in Section 5, which can then be leveraged to place and maintain the beam focus. Thus, localization and communication become inherently interleaved.

Notice that CSI acquisition is also closely tied to the specific technology employed to implement ELAAs. Indeed, conventional channel estimation requires the ability to access the CSI values individually for each antenna and RF chain. When different implementations are used (e.g., as previously mentioned, holographic surfaces, DMAs, dynamic arrays, or hybrid architectures), the channel acquisition procedure itself must be adapted to the underlying technology. In this context, leveraging the typical near-field localization properties can provide a practical mean to obtain the desired channel information without requiring explicit access to individual RF chains. As example, in [65] a sensing-assisted channel estimation method that reduces the number of required baseband samples and the codebook size due to the exploitation of the estimated location from sensing is proposed. Hence, beam management should be localization-aware: the uncertainty on the estimated position (e.g., ranging error) directly translates into defocusing, and, thus, motivates robust focusing strategies that trade peak gain with a larger focal region when position uncertainty is high (e.g., during fast maneuvers or partial blockage). Conversely, once accurate short-range localization is available (e.g., in platooning), aggressive focusing can be used to maximize spatial reuse and suppress interference toward unintended neighbors.

#### Numerical Example

It has been shown that beamfocusing can be effectively achieved as long as the user to be served is located at a distance *r* not exceeding one tenth of the Fraunhofer distance $r_{\mathrm{ff}}$ [53] when in the broadside direction of the array. Beyond this limit, the beam inevitably extends to infinity, similar to conventional far-field beamforming. For simplicity, let us consider a user satisfying this condition. The beam depth, defined as the spatial extent of the beam along this direction for a given threshold (e.g., −3 dB) with respect to the maximum value (i.e., the focal point), can be expressed as [56]

(4) $$\mathrm{BD}=\left\{\begin{array}{cc} \frac{20\;r_{\mathrm{ff}}\;r^{2}}{r_{\mathrm{ff}}^{2}-100\;r^{2}}, & r<\frac{r_{\mathrm{ff}}}{10},\\ \infty , & r\geq \frac{r_{\mathrm{ff}}}{10}. \end{array}\right.$$

From (4), it can be observed that the beam depth depends on both the Fraunhofer distance $r_{\mathrm{ff}}$ and the user distance *r*. In particular, an increase in the Fraunhofer distance results in a reduction in the beam depth as near-field effects become more pronounced.

**Figure 2:**
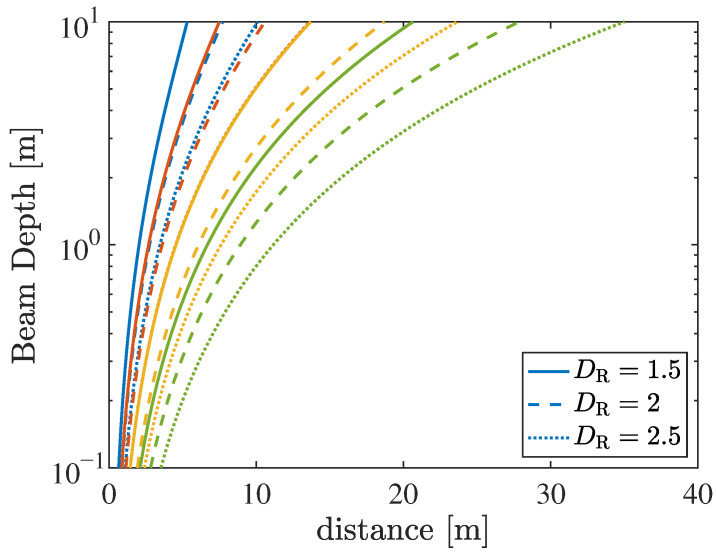
Beam depth as a function of the TX-RX distance for different array apertures. Different colors refer to different frequency bands (blue: FR1 at 5.9 GHz, red: FR3 at 10 GHz, yellow: FR2 at 28 GHz, and green: FR2 at 60 GHz).

Figure 2 illustrates the beam depth, as defined in (4), along the broadside direction of the transmitting array. This represents the most favorable condition, where near-field effects are most pronounced. Specifically, the figure shows how the beam depth varies as a function of the focusing distance *r*, i.e., the location where the intended vehicular user is assumed to be located. Different colors correspond to different frequency bands (blue: FR1 at 5.9 GHz, red: FR3 at 10 GHz, yellow: FR2 at 28 GHz, and green: FR2 at 60 GHz). For each frequency band, three different transmit ELAA apertures are considered (solid lines: $D_{T}=1.5$ m, dashed lines: $D_{T}=2$ m, and dotted lines: $D_{T}=2.5$ m). The same frequency–aperture combinations were previously adopted in Table 1 and Table 2 to define the corresponding Fraunhofer distances and the number of array elements.

It can be observed that, for all considered frequency bands, the beam depth becomes extremely small at very short distances from the transmitting array (e.g., 1–4 m). In this regime, the focusing spot exhibits a radial depth on the order of decimeters or even centimeters, resulting in very steep curves. On the one hand, this highlights the ability of large-aperture arrays to strongly focus the signal at very short ranges, thereby significantly reducing interference toward unintended users. On the other hand, this behavior also introduces critical challenges. For example, even a small displacement from the focusing point would rapidly cause a decrease in the beamforming gain, leading to a drastic reduction in the received power. Conversely, for more typical distances (on the order of several tens of meters), as may occur in vehicular platooning scenarios, the beam depth reaches values of about one meter, which is particularly attractive for practical vehicular applications. As the distance increases, the beam depth rapidly grows and eventually tends to infinity as the focusing distance approaches the Fraunhofer distance. Indeed, as previously discussed, at a distance of approximately $r_{\mathrm{ff}}/10$, the beam begins to extend indefinitely, resembling conventional far-field beamforming behavior. The beam depth decreases with increasing array aperture and carrier frequency as near-field effects become more pronounced.

Examples of near-field beamfocusing design strategies can be found in [66,67,68]. In [66], a near-field beamfocusing design is investigated in a wideband multi-user scenario, considering a hybrid beamforming architecture. Two algorithms based on true-time delayers are presented: a fully digital approximation approach and a heuristic two-stage approach. Regarding the first method, the hybrid beamformer is optimized by introducing a penalty method to approximate the optimal digital beamformer, guaranteeing convergence of the the spectral efficiency maximization model. The second method, instead, consists of a first step in which the analog beamformer is designed based on the maximization of the LOS signal power at each user, and subsequently, the digital beamformer is optimized by maximizing spectral efficiency, ensuring lower complexity. The work in [67] exploits a similar two-step approach, proposing a hybrid beamfocusing design in a near-field ISAC scenario, by considering a point target and an extended target. The goal is to optimize the Cramér–Rao lower bound (CRLB) for the point target and the Bayesian CRLB for an extended target. During the first step, an equivalent fully digital beamfocusing design is implemented, deriving a sub-optimal solution using the successive convex approximation technique. Subsequently, a block coordinate descend algorithm is employed to approximate the solution while considering a hybrid architecture. Lastly, the authors of [68], instead, propose a beamfocusing design method considering an ISAC system, with the aim of maximizing the minimum beam pattern gain. By exploiting semi-definite relaxation, a low complexity sub-optimal solution can be derived while still maintaining high performance.

### 4.2. LOS-MIMO

When both the transmitting and receiving vehicles are equipped with multiple antennas and operate within the near-field (Fresnel) region, the wireless channel exhibits fundamentally different properties compared to conventional far-field MIMO systems. In particular, near-field propagation enables the exploitation of multiple spatial DoFs even under pure LOS conditions and in the absence of rich scattering. This represents a major departure from classical far-field MIMO theory, where spatial multiplexing typically relies on multipath propagation to ensure a channel rank larger than one [27].

In the near-field regime, the channel between a transmit array and a receive array must be modeled using spherical wavefronts, and the distance between each transmit–receive antenna pair must be explicitly taken into account. The corresponding near-field phase distribution gives rise to multiple orthogonal *communication modes* supported by the propagation channel between the two arrays [5]. Consequently, the near-field LOS channel matrix generally exhibits a rank greater than one, enabling the transmission of multiple independent data streams simultaneously. Thus, unlike far-field LOS channels, near-field LOS channels support multiple modes associated with distinct spatial field distributions between the transmitter and receiver. In fact, each mode corresponds to a distinct spatial pattern of the field distribution between the two arrays, allowing multiple signals to be conveyed concurrently over the same time–frequency resources.

Let $H\in C^{N_{R}\times N_{T}}$ denote the near-field MIMO channel matrix between a transmit array with $N_{T}$ antennas and a receive array with $N_{R}$ antennas. Specifically, the element in position $(n^{r}$,$n^{t})$ of the matrix is proportional to $e^{-j\frac{2\pi }{\lambda }d\left(n^{r},n^{t}\right)}$, where $d(n^{r},n^{t})$ is the distance between the $n^{t}$-th transmit antenna and the $n^{r}$-th receive antenna. In the LOS near-field regime, H admits the singular value decomposition (SVD) [69]

(5) $$H=U\Sigma V^{H}\;,$$

where the diagonal matrix $\Sigma =\mathrm{diag}\left(\sigma_{1},\sigma_{2},\ldots ,\sigma_{I}\right)\in C^{N_{R}\times N_{T}}$ contains multiple, specifically *I*, non-negligible singular values. Each singular value corresponds to an independent communication mode. To fully exploit the available communication modes, appropriate signal processing must be performed at both the transmitter and the receiver. Specifically, a linear precoder based on the right singular vectors $V\in C^{N_{T}\times N_{T}}$ is applied at the transmitting vehicle, while a corresponding linear combiner based on the left singular vectors $U\in C^{N_{R}\times N_{R}}$ is employed at the receiving vehicle [69]. Formally, the transmit signal vector across antennas, with elements $x_{n}\left[k,m\right]$, for k=0,…,K−1 and m=0,…,M−1, is given now by $x\left[k,m\right]=Vs\left[k,m\right]\in C^{N_{T}\times 1}$, where $s\left[k,m\right]\in C^{N_{T}\times 1}$ is the data symbol. By exploiting the combining matrix $U^{H}$ at the receiving side, the MIMO channel is diagonalized, effectively transforming it into a set of *I* parallel, non-interfering subchannels. Thus, this operating principle differs from near-field beamfocusing techniques involving single-antenna receivers. While beamfocusing primarily exploits near-field effects to achieve power focus and interference suppression via spatial focusing, near-field MIMO leverages the same physical phenomena to enable spatial multiplexing. In this case, multiple antennas are not used solely to increase the beamforming gain; they also support the simultaneous transmission of multiple (i.e., *I*) data layers. As link with the beamfocusing discussed in Section 4.1, in [54] it has been shown that the number of communication modes (i.e., the channel rank) equals the number of orthogonal focused beams that the transmitting array can realize on the receiving aperture.

Spatial multiplexing is a cornerstone of MIMO communications, as it enables a linear increase in channel capacity with the number of independent spatial streams. In fact, the capacity of a MIMO channel is given by [69]

(6) $$C=\sum_{i=1}^{I}\log_{2}\left(1+\frac{p_{i}\sigma_{i}^{2}}{N_{0}}\right),$$

where *I* is the rank of the channel, $\sigma_{i}^{2}$ represents the squared singular values of H, $p_{i}$ denotes the power allocated to the *i*-th spatial mode, and $N_{0}$ is the noise power. The optimal power allocation across the modes is achieved through the well-known waterfilling strategy, which assigns more power to stronger modes while possibly shutting down weaker ones. A channel rank greater than one is favorable in the high-signal-to-noise ratio (SNR) regime. When the singular values are relatively balanced, as can occur in near-field LOS-MIMO configurations, the use of multiple modes can be particularly beneficial. In this case, power is distributed more evenly across the communication modes, and each subchannel contributes significantly to the overall capacity.

**Figure 3:**
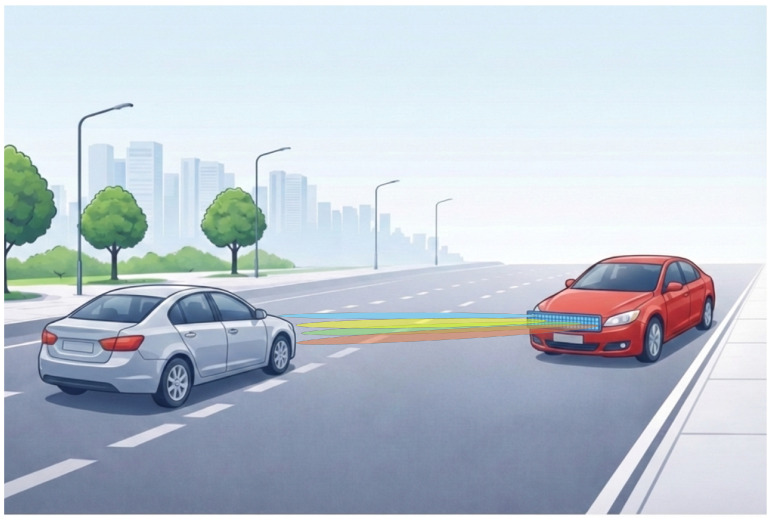
Pictorial example of communication in the V2X scenario using multiple orthogonal communication modes under the LOS condition, thanks to the use of ELAAs.

In near-field LOS scenarios, the availability of multiple communication modes enables the system to achieve substantial capacity gains over single-stream transmission. This is particularly relevant for vehicular communications, where high data rates, low latency, and reliable links are required for applications such as cooperative perception, sensor data sharing, and coordinated driving. Near-field LOS-MIMO is most impactful for short-range, high-rate V2V exchanges where the link is LOS-dominant and low latency is required; examples include sharing object lists/features during merges and supporting collective perception behind large occluders (trucks) on highways. Thus, vehicles equipped with large antenna arrays operating at high carrier frequencies can establish high-capacity links with nearby vehicles, even in open environments with limited multipath. In Figure 3, a near-field LOS-MIMO scenario is depicted, where the use of an ELAA on the front part of the two vehicles allows the emergence of multiple communication modes and, thus, the simultaneous transmission of multiple data streams.

On the other hand, near-field LOS-MIMO schemes (as with traditional SVD-MIMO) critically rely on accurate CSI at both the transmitter and the receiver to enable optimal precoding and combining. Imperfect or outdated CSI at either side results in a loss of subchannel orthogonality, leading to residual inter-stream interference and significant performance degradation. CSI estimation becomes particularly challenging in high-mobility scenarios or in the presence of strong Doppler effects, where the channel coherence time is severely reduced. Moreover, when channel estimation is performed on a single vehicle, the resulting CSI must be exchanged among communicating nodes, introducing additional latency and signaling overhead. In this context, the interplay between multiple frequency bands (such as FR1 for low-rate control/signaling, including CSI exchange, and FR2/FR3 for high-rate V2V communication) emerges as a promising solution. Similar to beamfocusing techniques, channel estimation can be significantly simplified by exploiting the interplay with localization. In fact, the knowledge of the relative positions of the vehicles (i.e., of the ELAAs) allows the definition of suboptimal yet practical precoding and combining strategies [54]. However, it is important to underline that, in this case, the relative orientation of the antenna arrays must be estimated, as it plays a fundamental role in defining the precoding/combining vectors. Consequently, joint position and orientation estimation becomes a key enabler for robust and efficient LOS-MIMO operations.

#### Numerical Example

Consider two vehicles equipped with ULAs, placed parallel to each other and aligned along their centers (i.e., in a paraxial configuration, typical when vehicles travel on the same lane). The apertures of the arrays are denoted as $D_{T}=N_{T}\delta$ and $D_{R}=N_{R}\delta$, and the distance between their centers is *r*. It can be demonstrated that the *I* most significant singular values in this configuration are nearly identical in magnitude and then rapidly decay [70]. Therefore, according to the waterfilling principle, power can be evenly distributed among the *I* modes. The number of DoFs of the link or the number of significant singular values *I* can thus be determined (for a single polarization) as [70]

(7) $$I=\frac{D_{T}D_{R}}{\lambda \;r}\;.$$

This classical expression, while accurately determining the number of DoF when $D_{T},D_{R}\gg r$ and the sizes $D_{T}$ and $D_{R}$ are nearly comparable, becomes inadequate when one of the apertures grows excessively large. In fact, (7) appears to suggest that the DoF can increase indefinitely if either $D_{T}$ or $D_{R}$ becomes infinitely large. However, the maximum number of DoFs is necessarily bounded. In fact, a transmitting array with $N_{T}$ antennas can realize at most $N_{T}$ orthogonal beams, leading to a maximum of $2D_{T}/\lambda$ DoFs obtained with half-wavelength spacing. Even an infinitely large receiving array cannot capture more than these $N_{T}=2D_{T}/\lambda$ beams, which is the maximum allowed number of communication modes even for $D_{R}\to \infty$, as discussed in [54]. A more accurate expression was derived in [54] by analyzing the scalar Green’s operator and modeling the communication modes as focused beams, which leads to

(8) $$\begin{array}{c} I=\frac{2D_{T}}{\lambda }\zeta \;, \end{array}$$

with $\zeta =D_{R}/\sqrt{4r^{2}+D_{R}^{2}}$. From Expression (8), it can be observed that as $D_{R}\to \infty$, we have ζ→1, and thus the limit is $I=\frac{2D_{T}}{\lambda }$, which corresponds to the actual limit (i.e., the available number of half-wavelength-spaced antennas). Among the $\frac{2D_{T}}{\lambda }$ orthogonal beamsteering directions that can be generated with the transmitting array of aperture $D_{T}$, (8) indicates the number of beams that intersect the parallel receiving array of aperture $D_{R}$ that is placed at a distance *r*.

**Figure 4:**
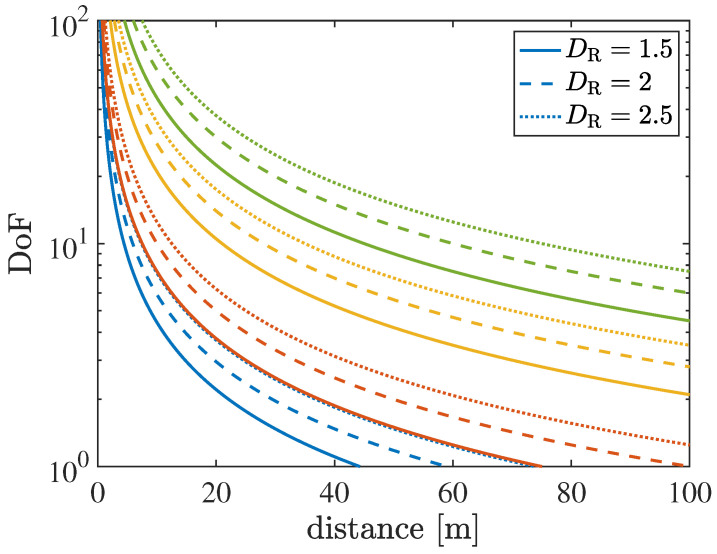
Number of communication modes as a function of the TX-RX distance for different array apertures. Different colors refer to different frequency bands (blue: FR1 at 5.9 GHz, red: FR3 at 10 GHz, yellow: FR2 at 28 GHz, and green: FR2 at 60 GHz).

Figure 4 illustrates the number of communication modes (i.e., the DoF for LOS-MIMO communications), as defined in (7). Similar to the beamfocusing case, the figure shows how the DoF varies as a function of the TX-RX distance *r*. Both the transmitting and receiving vehicles are assumed to be equipped with ELAAs. On the one hand, a fixed transmit aperture of $D_{T}=1.5$ m is considered, while on the other hand, three different receive apertures are assumed (solid lines: $D_{R}=1.5$ m, dashed lines: $D_{R}=2$ m, and dotted lines: $D_{R}=2.5$ m). Different colors correspond to different frequency bands (blue: FR1 at 5.9 GHz, red: FR3 at 10 GHz, yellow: FR2 at 28 GHz, and green: FR2 at 60 GHz).

It can be observed that the DoF decreases as the distance between the arrays increases. For instance, at a distance of 100 m and for FR3 with $D_{R}=2$ m, the number of available communication modes reduces to I=1. As the distance between the arrays decreases, the DoF increases very rapidly due to the interference among multiple propagation paths, which leads to an increase in the rank of the channel matrix H. For example, at an inter-array distance of 10 m, approximately I=4 communication modes are already available at FR1 for $D_{R}=1.5$ m, and the number of modes can increase up to approximately one hundred at FR2 at 60 GHz.

This behavior highlights how the channel capacity can dramatically increase at short inter-vehicle distances when large apertures are employed. This capability complements near-field beamfocusing techniques and provides a powerful tool for scaling throughput and spectral efficiency in specific vehicular scenarios, for example, when a couple of close-by vehicles need to exchange a large amount of data (e.g., raw sensor data for collective perception applications). Similar to the beamfocusing case, the DoF increases when the array aperture and carrier frequency increase, since near-field effects become more pronounced under these conditions.

## 5. ELAA for V2X Localization and Sensing

This section investigates how near-field propagation effects can be exploited for localization and sensing in vehicular scenarios, with a particular emphasis on ISAC systems.

The use of ELAAs operating in the near-field (Fresnel) region fundamentally changes the principles of wireless localization. Unlike conventional far-field systems, where localization is primarily angle-based and range estimation requires wideband signaling and eventually time synchronization, near-field propagation enables the extraction of distance information directly from the spatial structure of the received signal (as well as of other kinematic parameters, as will be discussed).

This section presents the key principles of near-field localization in vehicular scenarios, distinguishing between active localization and passive localization.

In Section 5.1, near-field techniques are discussed in the context of single-anchor localization, where the position of a transmitting vehicle can be determined by a receiving vehicle equipped with an ELAA. Moreover, the same principles are applied to near-field radar sensing, where a vehicle operating in a transmit–receive configuration estimates the position of another vehicle or object based on the reflected signal, highlighting the dual-use nature of near-field processing for communication and sensing.

In Section 5.2, the role of ELAAs in enabling enhanced Doppler analysis is analyzed. Compared to small arrays, ELAAs provide increased spatial resolution and sensitivity to motion-induced phase variations, allowing the separation of multiple Doppler components and the estimation of different velocity vectors. This additional kinematic information can be exploited for advanced functionalities such as predictive beamforming, target tracking, and proactive resource allocation in highly dynamic vehicular environments.

### 5.1. Single-Anchor Near-Field Localization and Sensing

#### 5.1.1. Near-Field Active Localization

In the active localization setting, a single-antenna transmit vehicle emits a known signal (e.g., a pilot in an OFDM frame) according to (2) for N=1, while an ELAA at the receive vehicle composed of $N_{R}$ antennas observes the impinging wavefront. When the transmitter lies within the Fresnel region of the receiving array, each antenna element experiences a distinct propagation distance, resulting in phase profile varying non-linearly across the array. By analyzing this nonlinear phase profile, the receiving array can jointly estimate both the AoA and the distance of the transmitter [71,72]. While angle estimation is also possible in far-field systems, where the phase profile is approximately linear, the ability to infer distance from the spatial phase curvature is a unique feature of near-field propagation. As a consequence, near-field localization enables single-anchor localization [73], where the position of a transmitter can be estimated using a single snapshot of received data and a single receiving array.

Formally, assume that the receiver is organized as a uniform linear ELAA, a reference system is placed with the origin corresponding to the center of the ELAA, and the position of the transmitter is $p={\left[\theta ,r\right]}^{T}$, where θ denotes the angle with respect to the broadside direction of the receiving ELAA (i.e., the AoA of the signal at the receiver) and *r* denotes the distance between the transmitter and the central element of the receiving ELAA. The useful component of the received signal $r\left[k,m\right]\in C^{N_{R}\times 1}$, for k=0,…,K−1 and m=0,…,M−1, with elements $r_{n}\left[k,m\right]$ can be written as (since the focus here is on near-field behavior, we neglect variations among the different subcarriers by assuming small bandwidth)

(9) $$r_{n}\left[k,m\right]=\sqrt{P}\beta \;e^{-j2\pi f_{c}\tau_{n}\left[p\right]}e^{j2\pi \nu_{n}mT_{s}}e^{j\varphi }$$

where *P* is the transmit power, φ accounts for the phase synchronization mismatch between the transmitter and the receiver, β is the channel scaling coefficients, $\nu_{n}$ denotes the Doppler shift at the *n*-th receiving antenna, and the term $\tau_{n}\left[p\right]=r_{n}\left[p\right]/c$ denotes the time taken by the signal to travel between the transmitter and the *n*-th receiving antenna at distance $r_{n}$. The channel scaling coefficient is given by the Friis law and can be written as

(10) $$\beta =\sqrt{\frac{G_{T}G_{R}\lambda^{2}}{{\left(4\pi r\right)}^{2}}}\;,$$

where $G_{T}$ and $G_{R}$ are the transmitting and receiving antenna gains.

A remarkable aspect of near-field localization is that distance estimation can be achieved even with narrowband signals by processing the antenna-dependent phase profile in (9). In classical far-field systems, range estimation typically relies on wideband waveforms to extract time-of-arrival (ToA) information from the frequency-dependent phase across multiple subcarriers. Such approaches require either sub-nanosecond synchronization between the transmitter and the receiver, differential techniques such as time-difference-of-arrival (TDoA) estimation, or two-way ranging (TWR) protocols. These solutions are often complex, inefficient in terms of channel usage (e.g., TWR), or difficult to implement in highly mobile vehicular environments. In contrast, near-field localization exploits the spatial distribution of the phase across the array aperture rather than its frequency dependence. As a result, range information can be extracted from a single narrowband transmission, without the need for tight synchronization or multiple signaling rounds. This enables single-shot localization, which is particularly attractive in fast-changing scenarios.

**Figure 5:**
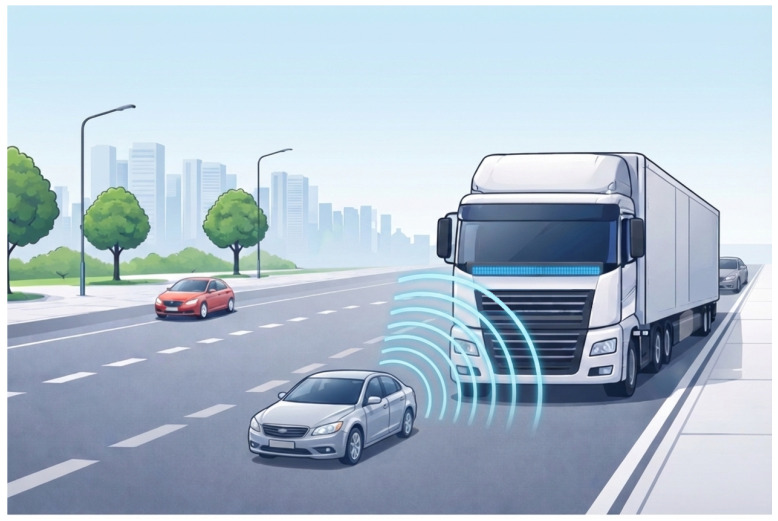
Pictorial example of a platooning scenario, where the vehicle equipped with the ELAA localizes the transmitting single-antenna vehicle by the analysis of the spherical received wavefront.

However, the quality of near-field range estimation strongly depends on the distance between the transmitter and the receiving array. As the distance increases, the curvature of the wavefront becomes weaker, and the received signal gradually approaches a planar-wave structure. Consequently, the estimation accuracy for the range information degrades as the transmitter moves toward the far-field region. This characteristic makes near-field localization especially suitable for short-range scenarios, for example, vehicle platooning or close-proximity sensing, where vehicles operate at distances well below the Fraunhofer boundary [37]. An example of vehicle platooning is shown in Figure 5, where the position of the single-antenna vehicle is inferred by the truck equipped with the ELAA exploiting the nonlinearity of the phase profile, which is enabled by the proximity between the two vehicles.

Near-field V2X localization thus enables a single-anchor localization paradigm, offering several key advantages that are particularly relevant in vehicular scenarios. First, it provides *high accuracy*, which naturally increases as the inter-vehicle distance decreases, i.e., in those situations where stringent accuracy requirements are most critical, such as for safety-critical interactions (e.g., platooning). Conversely, at larger distances, the localization accuracy requirements can be relaxed. Second, the proposed approach guarantees *high availability*, since positioning information is obtained independently of the coverage of fixed infrastructures or satellite-based systems. As a result, no external anchor-based localization infrastructure is required beyond the vehicles themselves; however, this also implies that only relative vehicle positions are estimated, rather than absolute coordinates, as in GNSS-based solutions. Third, it enables a *high update rate*, as no multi-sensor data fusion or cooperation with additional anchors is needed. A new position estimate can be generated at each received signal instance (e.g., at every pilot sequence within a data packet), allowing position updates several times per second and effectively supporting real-time vehicular control systems, such as automated driving engines. Fourth, the approach achieves *low latency*, since position estimates are immediately available without interactions with other vehicles or infrastructure nodes or multi-user signal processing. This allows for timely reactions even in high-speed scenarios. Finally, it does not require tight synchronization among vehicles or infrastructure, which may be difficult to guarantee in practice. The reuse of communication signals for localization purposes, for instance through embedded pilot symbols, further ensures minimal additional spectrum consumption.

#### 5.1.2. Near-Field Passive Localization

The same principles underlying active near-field localization can be extended to passive localization, where the objective is to estimate the position of a target rather than a transmitting node. In this case, the system operates as a radar or as an ISAC platform. A transmitting array illuminates the environment, and the receiving ELAA processes the signal reflected by a target, such as another vehicle [74].

In near-field radar operation, the reflected signal exhibits a spatial phase profile that depends on both the angle and the distance of the target. As in the active case, this allows joint angle and range estimation from the spatial structure of the received signal, potentially with narrowband waveforms. However, several additional challenges arise. First, the propagation path experiences an increased path loss due to the round-trip transmission, and the received power depends on the radar cross-section of the target. Second, targets in near-field vehicular scenarios are often spatially extended rather than point-like, which complicates modeling and parameter estimation [75,76,77].

Formally, assume the same geometry of the active localization case, but with the target placed in $p={\left[\theta ,r\right]}^{T}$, where θ denotes the angle with respect to the broadside direction of the receiving ELAA (i.e., the AoA of the signal at the receiver) and *r* denotes the distance between the target and the central element of the receiving ELAA. The useful component of the signal received after the reflection of the target can still be written according to (9) by considering that, now, $\tau_{n}\left[p\right]=d+r_{n}\left[p\right]/c$ denotes the sum of the time taken by the signal to travel between the transmitter placed at distance *d* from the target and the *n*-th receiving antenna at distance $r_{n}$. The channel scaling coefficient is given now by

(11) $$\beta =\sqrt{\frac{G_{T}G_{R}\lambda^{2}\rho }{{\left(4\pi \right)}^{3}d^{2}r^{2}}}\;,$$

where ρ is the radar cross-section (RCS) of the target.

When multiple antennas are used only at the receiver, near-field effects can still be exploited to estimate the target distance and angle. However, further gains can also be achieved by leveraging multiple antennas at the transmitter. In this case, the transmitted waveform can be spatially shaped to improve the SNR in the region where the target is expected to be located [78]. To exploit transmit-side spatial processing, some form of prior information about the target location is generally required. For instance, beamfocusing techniques can be used to concentrate the transmitted energy into a specific spatial region, thereby enhancing detection performance and reducing interference. This approach is particularly effective when the target is known to lie within a limited angular and distance range, such as in forward-looking automotive radar applications.

An alternative strategy is time-division beamforming, in which different spatial regions are sequentially illuminated by the transmitting array. The receiver then attempts to detect the presence of a target in each region. While this approach does not require precise prior knowledge of the target position, it can be inefficient in near-field scenarios. Since the near-field beamforming vector depends on both angle and distance, covering the entire search space may require a large number of spatial configurations. This can lead to long scanning times, which are problematic in vehicular environments characterized by fast mobility and rapidly time-varying channels. On the other hand, the localized focal region enables high-resolution spatial probing, allowing the detection and even separation of targets at different distances along the beam direction. As discussed in Section 4.1, the achievable focal beam depth and shape depend on the array aperture, carrier frequency, focal distance, and angle, highlighting the importance of carefully designing array parameters to exploit near-field effects effectively in practical vehicular deployments.

Even in vehicular sensing applications, radar systems can be broadly classified into monostatic and bistatic configurations, depending on the relative positions of the transmitter and receiver. In a monostatic radar system, the transmitting and receiving antennas are co-located on the same vehicle, illuminating the environment and processing the echoes reflected by surrounding targets. This configuration is widely adopted in automotive radar systems due to its simplicity and tight integration with on-board sensors, enabling straightforward estimation of target range, angle, and velocity. Note that implementing monostatic sensing necessitates the use of a full-duplex radio system. In contrast, bistatic radar systems involve spatially separated transmitters and receivers, which may be located on different vehicles or infrastructure nodes. In vehicular environments, bistatic operation enables cooperative sensing, where one vehicle transmits, while another receives the reflected signal, providing additional spatial diversity and improving target observability. Two main challenges must be considered for the implementation of bistatic/multistatic sensing: (i) the need for tight synchronization (e.g., at sub-nanosecond level) between the transmitting and receiving vehicles and (ii) the need for precise knowledge of the position of both TX and RX vehicles. While requirement (i) is only needed for TDoA-based sensing but not for near-field-based sensing, requirement (ii) is always needed for bistatic sensing, making it extremely challenging, especially in dynamic V2X contexts.

#### 5.1.3. Numerical Example

The maximum achievable estimation accuracy can be characterized through the CRLB [79]. This fundamental tool provides a lower bound on the variance of any unbiased estimator, thereby quantifying the minimum estimation error that can be attained given a specific signal model and the system geometry. As such, the CRLB represents a benchmark for performance evaluation, allowing one to assess how close a practical estimator operates with respect to the theoretical limit imposed by the underlying physical and statistical assumptions. When the transmitter is located in the near-field region of the receiving array, assuming a classical OFDM signal as in (2) and $N_{R}$ receiving antennas, the corresponding CRLB for distance estimation has the form [80]

(12) $${\mathrm{CRLB}}^{\left(r\right)}=\frac{6c^{2}r^{2}\left(\delta^{2}\left(N_{R}^{2}-4\right)\sin^{2}\theta_{R}+15r^{2}\right)}{\pi^{2}f_{c}^{2}MKN_{R}\;\mathrm{SNR}\;\delta^{4}\left(N_{R}^{2}-4\right)\left(N_{R}^{2}-1\right)\cos^{4}\theta_{R}}\;.$$

where *r* is the transmitter-array distance, $\theta_{R}$ is the AoA at the receiving array, and *M* and *K* are the number of OFDM symbols and subcarriers, respectively. Notice that a linear SNR gain $MKN_{R}$ due to the multiple observations coming from the OFDM symbols, subcarriers and antennas is experienced. As anticipated, it is interesting to observe that the accuracy of range estimation in (12) decreases as the target moves away (i.e., large *r*). Assuming a transmitter on the broadside direction of the array (i.e., $\theta_{R}=0$), (12) simplifies to

(13) $${\mathrm{CRLB}}^{\left(r\right)}=\frac{90\lambda^{2}r^{4}}{\pi^{2}MKN_{R}\;\mathrm{SNR}\;D_{R}^{4}}\;,$$

with $\delta^{4}\left(N_{R}^{2}-4\right)\left(N_{R}^{2}-1\right)\approx D_{R}^{4}$. From (13), it is clear that the fundamental figure of merit driving estimation accuracy is the distance-to-aperture ratio $r/D_{R}$. Increasing the carrier frequency with a fixed aperture is beneficial for improving the estimation accuracy (stronger near-field effects). Even in the radar case, the estimation quality in terms of CRLB is given by Expression (12). However, the SNR scaling changes drastically, as it involves two-way propagation, as determined by the radar range equation.

**Figure 6:**
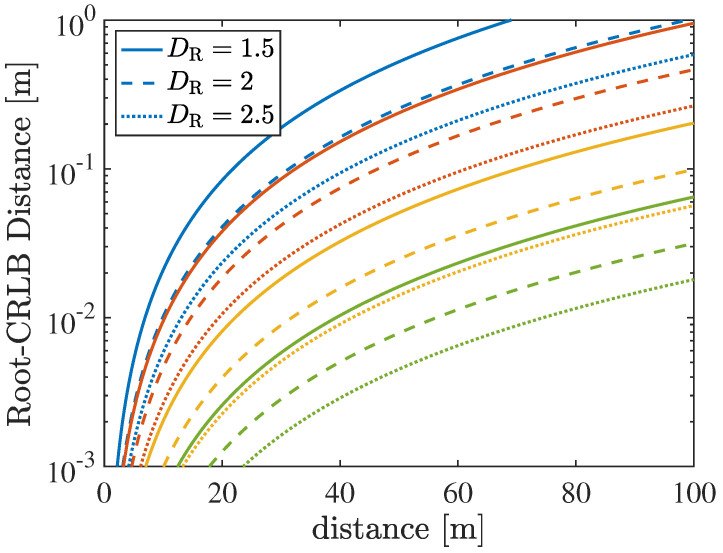
Root-CRLB for distance estimation for different array apertures. Different colors refer to different frequency bands (blue: FR1 at 5.9 GHz, red: FR3 at 10 GHz, yellow: FR2 at 28 GHz, and green: FR2 at 60 GHz).

Figure 6 reports the root-CRLB for distance estimation, as defined in (13). Specifically, the figure shows how the localization accuracy for distance estimation varies as a function of the TX-array distance, where the receiver is equipped with an array of aperture $D_{R}$. Three different apertures are considered (solid lines: $D_{R}=1.5$ m, dashed lines: $D_{R}=2$ m, and dotted lines: $D_{R}=2.5$ m). Different colors correspond to different frequency bands (blue: FR1 at 5.9 GHz, red: FR3 at 10 GHz, yellow: FR2 at 28 GHz, and green: FR2 at 60 GHz). The SNR is fixed to 0 dB. We consider M=14 OFDM symbols and K=128 subcarriers. The performance naturally scales with the number of symbols and subcarriers employed due to the corresponding increase or decrease in the number of independent observations available for distance estimation. Concerning the ELAA, an inter-element spacing of δ=λ/2 is assumed.

The CRLB is observed to increase as the distance between the arrays increases. This behavior represents a key characteristic of near-field-based distance estimation techniques, in contrast to traditional wideband ranging methods, for which the CRLB does not depend on the parameter to be estimated, namely the distance itself. At short distances from the receiving array, the estimation accuracy is extremely high (i.e., a low CRLB) thanks to the strong near-field conditions. Indeed, distance estimation can be interpreted as the dual problem of the beamfocusing mechanism previously discussed. In the same way that a transmitting ELAA can focus the beam with a very small beam depth at short distances, a receiving ELAA can achieve highly accurate distance estimation at close range by analyzing the spherical wavefront impinging on the array. As the distance between the transmitter/target and the receiving ELAA increases, the estimation accuracy progressively deteriorates. This approach is therefore particularly appealing for platooning scenarios, where the short inter-vehicle distances enable extremely accurate localization. More generally, estimation accuracy degradation with distance can be tolerated in many practical applications, since larger localization errors are typically acceptable as the distance between vehicles increases.

In a real system, the SNR is not a fixed parameter but rather depends on the distance itself; in this sense, it can vary significantly depending on whether the estimation is performed in an active TX-array mode or in a passive (radar-like) TX-target-array configuration. Since the array aperture represents the main figure of merit driving estimation accuracy, a possible approach to simplify system implementation could consist of employing large arrays with an inter-element spacing larger than λ/2. While this strategy preserves a large physical aperture and thus allows near-field effects to be exploited, it also entails a loss due to the reduced SNR gain, which is caused by the smaller number of independent observations. Recent works have also shown that, in low-SNR regimes, ambiguities in position estimation may arise [81]. Moreover, it should be noted that when traditional V2X systems operating at 5.9 GHz are employed, especially for broadcast transmissions, interference can lead to significant performance degradation, as analyzed in [37].

Several near-field localization and sensing algorithms are proposed and investigated in the literature. Some possible solutions are reported hereafter. In [82], the authors introduce a method to reduce the complexity of the multiple signal classification (MUSIC) algorithm by partitioning the directional matrix into the directions of arrival and range. In this way, a single 1D spectral search is needed instead of the 2D search required in the standard MUSIC algorithm. As a different solution, the work in [83] is based on the principle that partitioning the ELAA into several subarrays allows us to consider the user in the far-field region. Therefore, by exploiting the geometric relationships between the user location and the AoAs at each subarray, a probabilistic near-field localization problem is derived. The proposed algorithm starts from this probabilistic model and then performs message passing on a factor graph, which represents the signal model for each subarray under far-field conditions. This location estimate is then refined by performing maximum likelihood estimation. In [84], an algorithm is proposed for the joint estimation of position and velocity of a moving point target, two quantities that are tightly coupled in the near field. It follows a similar approach as in [83], considering a subarray-based variational message passing method based on a factor graph representation. This allows decoupling between position and velocity parameters. By processing all the subarray measurements, centimeter-level localization and sub-m/s velocity accuracy is achieved while reducing computation complexity.

### 5.2. Predictive Beamforming

Beam alignment between two moving vehicles is one of the most complex challenges in mmWave communications [17,85,86]. Due to the directional nature of the beams and the rapid position changes in the vehicles, the system must dynamically update the beam direction to maintain a stable connection. Several techniques can be conceived, for example:

1. *Beam Sweeping*: Vehicles transmit and receive signals in multiple angular directions to determine the optimal beam alignment. This process is similar to what is used in 5G networks, where the transmitter and receiver test various angles until they find the best one. However, it is a relatively slow process and may not be suitable for high-speed vehicle scenarios or when large arrays (i.e., numerous narrow beams) are adopted.
2. *Beam Tracking*: After the initial beamsweeping phase, the system continuously monitors position changes and dynamically updates the beam direction. Techniques such as periodic receiver feedback or predictive filtering (e.g., Kalman Filter) can enhance accuracy.
3. *Predictive Beam Steering*: This method exploits navigation data and on-board sensors, including the global positioning system (GPS), the inertial measurement unit (IMU), LiDAR, radar, and cameras, to predict the vehicle trajectory and proactively estimate the optimal beam direction. By leveraging machine learning- and AI-based algorithms, the system dynamically adapts to lane changes, road curvature, and variations in vehicle acceleration [87].
4. *Side-Information-Assisted Beamforming*: In addition to the GPS and sensors, vehicles can exchange state information (speed, acceleration, and direction) to improve beam alignment. This approach reduces the time required to find the optimal beam.

When ELAAs are employed, maintaining a focused beam alignment becomes even more challenging. In this case, alignment must be ensured not only in the angular domain but also in the radial domain. On the other hand, while this requirement may initially represent an additional complication, the use of ELAAs enables novel predictive beamforming strategies capable of simplifying beam alignment and tracking. These strategies are made possible by exploiting the non-uniform Doppler characteristics observed across the different antenna elements composing the array according to the model in (9) [88,89]. In fact, in near-field conditions enabled by ELAAs, both the radial and transverse velocity components can be extracted to obtain a full picture on relative mobility [90]. Specifically, transverse velocity estimation is made possible by the proximity to the array, thus enabling the projection of the velocity vector along the set of directions between a target/transmitter and each receiving antenna element [91]. In far-field conditions, instead, the array can be seen practically as a point, thus obtaining the same projection on all the antennas; in this case, only the radial component of the velocity can be retrieved. Therefore, radial and transverse velocity components are estimated together with distance and angle thanks to the near-field condition, allowing full knowledge of the target/transmitter mobility status and thereby providing pivotal information to perform predictive beamforming [92]. As a consequence, no prior information on the trajectory shape is needed, which is different from what happens in the far field.

**Figure 7:**
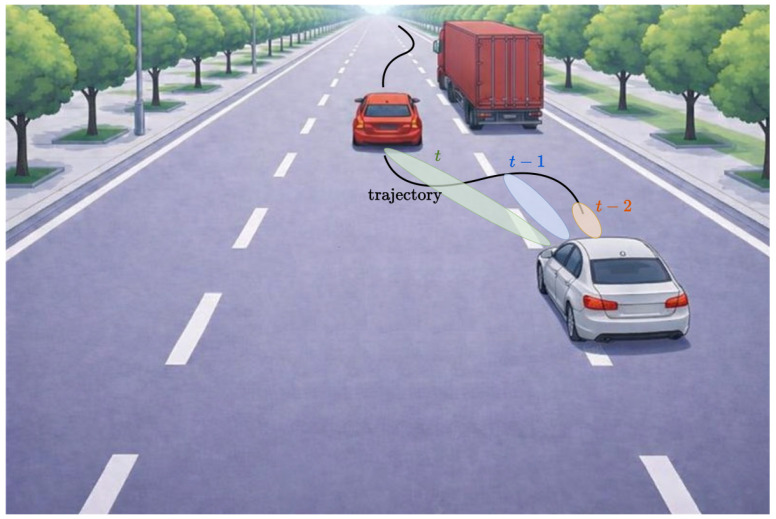
Pictorial example of an ELAA-equipped vehicle performing near-field predictive beamforming by estimating the 2D velocity vector and tracking the evolution of the vehicle position.

In the passive case, suppose a vehicle aimed at transmitting a signal to a receiving vehicle adopting beamforming techniques (even angle-based beamsteering and/or beamfocusing if an ELAA is adopted). By resorting to ISAC, suppose that such a transmitting vehicle performs jointly monostatic radar operations, thus obtaining an estimation of the relative position and velocity of the receiving vehicle and corresponding to the radar target. Thanks to the use of ELAAs for ISAC, the 2D velocity is estimated, thereby acquiring the full picture of the relative velocity vector in the plane. As the transmitting vehicle estimates the position and velocity of the vehicle it wants to communicate with using radar, it can use these estimates to update the kinematic model and predict the next beam accordingly. This is shown in Figure 7, where the gray car performs predictive beamforming toward the red car that is overtaking. The beams at different time instants are indicated to emphasize the correspondence between the evolution of the gray car’s positions and the predicted beams.

A different scheme can also be devised in the active case, without resorting to ISAC (i.e., radar). Suppose that both vehicles are equipped with ELAAs and communicate (thus, the respective beams are aligned/focused). At a given time slot, one vehicle transmits data by pointing the beam toward the vehicle it wants to establish the communication with. The receiving vehicle can then perform Doppler estimation along its receiving ELAA, as it is able to retrieve the velocity vector, as occurs in the passive case. This information can then be exploited in the subsequent time slot if the receiving vehicle aims to respond with a transmitted packet. Each time an ELAA-equipped vehicle receives a data packet, it updates the kinematic model describing the relative motion of the transmitting vehicle. This information can then be exploited in subsequent transmission to adapt the beam in a predictive manner, without risking loss of alignment.

#### Numerical Example

As for distance estimation, the CRLBs for radial and transverse components are provided to identify the best achievable performance and, consequently, to grasp some insights into how system and geometric parameters affect estimation accuracy.

Specifically, the near-field CRLB for the estimation of the radial velocity $v_{r}$ is approximately equal to that of the far-field if the target is not too close to the array, and its expression is [91]

(14) $${\mathrm{CRLB}}^{\left(v_{r}\right)}=\frac{3c^{2}}{8\pi^{2}f_{c}^{2}MKN_{R}\;\mathrm{SNR}\;\left(M^{2}-1\right)T_{s}^{2}}\;,$$

where $T_{s}$ is the symbol time such that $\left(M^{2}-1\right)T_{s}^{2}\approx T_{\mathrm{obs}}^{2}$ is the square of the signal duration used for velocity estimation. Accordingly, the accuracy for radial velocity estimation increases by enlarging the observation interval, thereby improving the Doppler resolution. Moreover, similar to (12), the term $MKN_{R}$ represents the SNR gain experienced through multiple independent observations over different OFDM symbols, subcarriers, and antennas.

Concerning transverse velocity $v_{t}$, the corresponding CRLB along the broadside direction (i.e., $\theta_{R}=0$) is [91]

(15) $${\mathrm{CRLB}}^{\left(v_{t}\right)}=\frac{18c^{2}\;r^{2}}{\pi^{2}\;f_{c}^{2}\;MKN_{R}\;\mathrm{SNR}\;\left(M^{2}-1\right)T_{s}^{2}\left(N_{R}^{2}-1\right)\delta^{2}}\;.$$

As the AoA increases, the accuracy for transverse velocity estimation decreases accordingly, since when $\theta_{R}\to \pm \pi /2$, all the antenna elements see the incoming wavefront from a single direction corresponding to the radial direction. Therefore, only the radial component of the velocity can be estimated in this condition [91]. Since $\left(N_{R}^{2}-1\right)\delta^{2}\approx D_{R}^{2}$, (15) simplifies to

(16) $${\mathrm{CRLB}}^{\left(v_{t}\right)}=\frac{18\lambda^{2}\;r^{2}}{\pi^{2}\;MKN_{R}\;\mathrm{SNR}\;T_{\mathrm{obs}}^{2}D_{R}^{2}}\;.$$

As before, also for transverse velocity, the performance scales with the signal duration $T_{\mathrm{obs}}$. Unlike (14), here, the main figure of merit driving the estimation accuracy is the distance-to-aperture ratio $r/D_{R}$. Therefore, if the target moves far away from the receiving array or the antenna aperture is small, the estimation of the transverse component becomes unreliable. A higher carrier frequency is beneficial for both radial and transverse velocity estimation if a fixed aperture is considered. However, it is interesting to notice that, if a half-wavelength array is employed (i.e., δ=λ/2), the transverse velocity’s accuracy becomes insensitive to the carrier frequency. In fact, in this case, the aperture shrinks as the wavelength diminishes. Thus, the advantage of using a larger carrier for finer Doppler estimation is counterbalanced by the loss coming from using a smaller aperture.

**Figure 8:**
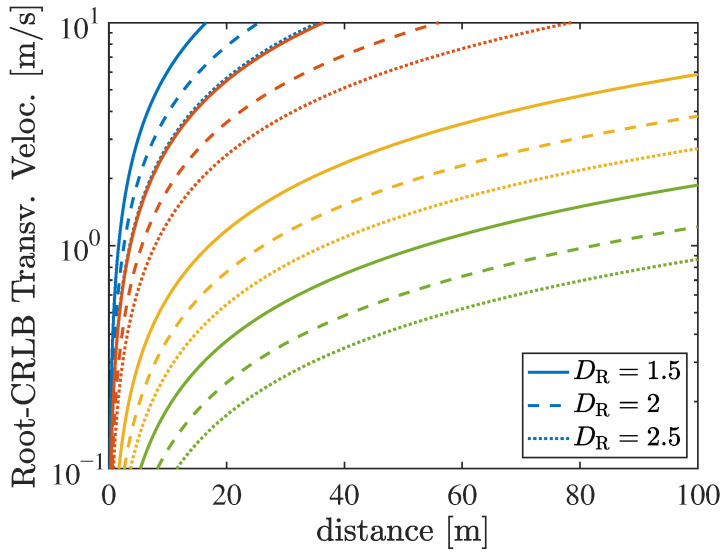
Root-CRLB for transverse velocity estimation for different array apertures. Different colors refer to different frequency bands (blue: FR1 at 5.9 GHz, red: FR3 at 10 GHz, yellow: FR2 at 28 GHz, and green: FR2 at 60 GHz).

Figure 8 reports the root-CRLB for the estimation of the transverse velocity of a target using a receiving ELAA, as defined in (16). Three different ELAA apertures are considered (solid lines: $D_{R}=1.5$ m, dashed lines: $D_{R}=2$ m, and dotted lines: $D_{R}=2.5$ m). Different colors correspond to different frequency bands (blue: FR1 at 5.9 GHz, red: FR3 at 10 GHz, yellow: FR2 at 28 GHz, and green: FR2 at 60 GHz). As in the previous analysis, the SNR is fixed to 0 dB. We consider M=14 OFDM symbols and K=128 subcarriers, with symbol time $T_{s}=16.6\;\mu s$, which are representative values for 5G-like OFDM systems. The performance naturally scales with the number of symbols and subcarriers employed due to the corresponding increase or decrease in the number of independent observations available for parameter estimation. Concerning the ELAA, an inter-element spacing of λ/2 is assumed. In this context, besides the array aperture, the main figure of merit affecting estimation accuracy is the symbol time, which directly affects the observation interval $T_{\mathrm{obs}}$; increasing this value improves the estimation performance. Clearly, the observation time must remain compatible with the target mobility and with the quasi-stationarity assumptions of the channel.

The CRLB is observed to increase as the distance between the transmitter and the receiving array grows. As already observed for distance estimation, this behavior represents a fundamental characteristic of near-field-based operations. Conversely, the accuracy of radial velocity estimation does not depend on the array aperture, as discussed in [90]. At short distances from the receiving array, the estimation accuracy is extremely high (i.e., the CRLB is very low) thanks to the strong near-field conditions. In this regime, each array element experiences a different projection of the velocity vector and, consequently, a different Doppler shift, enabling the analysis of the target motion from multiple spatial perspectives and allowing the reconstruction of the complete velocity vector in the plane. As the distance between the transmitter and the receiving ELAA increases, the estimation accuracy progressively deteriorates. It is worth noting that the estimation of the radial velocity component is always significantly more accurate, specifically by several orders of magnitude than that of the transverse velocity component, as discussed in [90]. The estimation of the transverse velocity thus represents the main bottleneck of Doppler-based predictive beamforming systems. Once a sufficiently accurate estimate of this component is obtained, it can be reasonably assumed that the full velocity vector is known with comparable accuracy. Based on this information and by extrapolating the motion of the vehicle hosting the array, it becomes possible to predict the future position of the target vehicle, thereby enabling anticipatory beamforming and preventing potential communication link outages.

Similar to distance estimation, in a realistic system, the SNR is not a fixed parameter but rather depends on TX-RX separation. Moreover, sparse arrays enabling large apertures can be more easily considered in this context, as ambiguities are less likely to arise. In all the considered applications, namely beamfocusing, LOS-MIMO, near-field localization, and Doppler-based predictive beamforming, paraxial configurations between arrays or scenarios where the vehicle/target is deployed along the broadside direction of the ELAA have been assumed, since near-field effects are most pronounced in this condition. In general, for all applications, performance degrades as the AoA $\theta_{R}$ increases. In the worst-case scenario (end-fire direction, $\theta_{R}=\pi /2$), near-field effects are no longer observable; consequently, distance estimation, beamfocusing, and transverse velocity estimation are not feasible. Alternative array configurations (e.g., circular arrays) or multiple arrays (e.g., mounted on different sides of the vehicle) may be envisioned to overcome these blind directions and enable angle-insensitive operation.

## 6. Conclusions and Future Research Directions

This paper has explored the potential of ELAAs deployed on board vehicles to enhance communication, localization, and sensing in 6G vehicular networks. By focusing on near-field signal processing, we analyzed how beamfocusing, LOS-MIMO, single-anchor localization and sensing, and Doppler-based predictive beamforming can jointly improve system performance in V2X sidelink scenarios. The main contributions of this work can be summarized as follows:

- Discussion of near-field beamfocusing in multi-user MIMO V2X communication, improving interference mitigation and spatial reuse.
- Analysis of LOS-MIMO in short-range vehicular links, quantifying achievable DoFs as a function of array size, carrier frequency, and inter-vehicular distance.
- Investigation of single-anchor localization using ELAA-equipped vehicles through both classical transmitter localization and radar-based sensing, methods particularly effective for platooning and short-range scenarios.
- Study of predictive beamforming via Doppler analysis on ELAAs, applicable to both direct communication and radar-based sensing, allowing anticipatory alignment of beams for dynamic vehicular scenarios.
- Examination of the impact of carrier frequency and array scaling on communication, localization, and sensing performance, covering the FR1, FR2, and FR3 frequency ranges.

Fundamental constraints stem from near-field physics: (i) the beam management problem involves inherently a polar domain (angle–range), which increases the dimension of the parameter space and the pilot overhead needed for reliable tracking under mobility, and (ii) focusing the signal at short distances implies a small beam depth, making performance sensitive to position uncertainty and requiring robust (wider) focusing when localization is imperfect. Current technological constraints are implementation-driven: (i) phase coherence across large distributed vehicular apertures (and its calibration/maintenance under temperature, vibration, and aging), (ii) limited RF-chain counts motivating hybrid architectures, and (iii) processing or energy budgets that constrain real-time operations for localization and communication. These limitations motivate the research directions below, including calibration-aware designs, localization-aided CSI reduction, and hardware–algorithm co-design for scalable ELAA-V2V ISAC.

While the results demonstrate the promise of ELAA-enabled near-field ISAC, several open research challenges remain. We identify and discuss key directions for future investigation below.

- **Implementation-related aspects:** Realizing extremely large arrays on vehicles involves significant structural and mechanical challenges. Vehicle surfaces are often non-flat, potentially requiring *conformal arrays*. Achieving $360^{\circ }$ coverage may require multiple arrays on different faces of the vehicle or circular arrays mounted on the roof. Circular arrays could also mitigate issues associated with null directions of linear arrays. Ensuring phase coherence across such large arrays is critical, and synchronization or positioning errors can significantly impact performance, especially at higher carrier frequencies. In addition, mutual coupling and vehicle-induced pattern distortion should be carefully considered. Research is needed to evaluate practical integration, robustness, and calibration procedures for in-field deployment. This also requires novel and ad hoc near-field V2X channel measurements and modeling.
- **CSI acquisition and signal processing:** Near-field communication and sensing with ELAAs pose significant signal-processing challenges. CSI acquisition presents several issues that are not encountered in conventional far-field scenarios. First, the acquisition process is strongly dependent on the underlying array technology: in implementations such as holographic surfaces, DMAs, dynamic arrays, or hybrid architectures, the CSI values may not be directly accessible for each antenna, requiring novel reconstruction strategies. High-resolution channel estimation needs extended codebooks and fine spatial sampling, which increases computational complexity. Joint estimation of channel state and relative position/orientation could provide efficiency gains, but it requires practical algorithms capable of handling the near-field regime. Specifically, joint multi-parameter estimation (e.g., range, angle, radial velocity, transverse velocity, orientation, etc.) in the near field (dealing with not only high-dimensional signals measured over large apertures but also wide bandwidths and long acquisition times) constitutes a challenging signal-processing problem that must be carefully addressed to ensure computationally feasible and practically implementable solutions. Efficient beam management, adaptive beamforming, and predictive processing leveraging motion and Doppler information with low-complexity solutions remain important open problems. Optimal waveform design for joint communication and multi-parameter estimation under near-field conditions represents another potential investigation area.
- **Hardware–algorithm co-design:** Ad hoc solutions are required to enable the practical realization of ELAAs while maintaining acceptable hardware complexity (e.g., number of RF chains) and energy consumption. In this context, sparse arrays, metasurface-based antennas, reconfigurable antennas, holographic surfaces, and hybrid array architectures should be systematically investigated to identify practical solutions for vehicular near-field operations across different frequency bands. A key challenge lies in achieving an optimal trade-off between processing performed at the hardware and software (baseband/algorithmic) levels, as well as in the electromagnetic domain.
- **Radar target modeling and processing:** Near-field ISAC operations may involve very short distances between the antenna arrays and the targets. In such scenarios, the classical point-target assumption is often violated. Consequently, a single object (e.g., a reflective vehicle) must be modeled as an extended target, which in turn requires appropriate modifications of the signal-processing algorithms employed for detection and parameter estimation.
- **Transmit power and regulatory constraints:** Regulatory limits typically involve equivalent isotropically radiated power (EIRP), thus imposing constraints on the per-element transmit power of ELAA systems. Since, near-field effects are more pronounced at high frequencies, where typically lower transmit power levels are used, the interplay among near-field benefits and available transmit power must be carefully investigated. Future research should explore power allocation strategies (even in the presence of spatial non-stationary conditions), energy-efficient designs, and techniques to maintain the required performance level while respecting regulatory constraints.
- **Electromagnetic exposure and safety:** Large arrays generate complex electromagnetic fields. Ensuring compliance with exposure limits, both out of the vehicle and in the vehicle, for human safety is crucial, particularly for the passenger compartments. Research is needed to quantify exposure, design arrays with minimal impact on occupants, and develop safe operational protocols.
- **Frequency allocation, interference, and resource management:** Different frequency bands present diverse propagation and interference characteristics. Understanding which services can be allocated to each band while considering broadcast versus unicast transmission is critical. Policy design is needed to enable high-data-rate communication with relevant vehicles while minimizing interference and to determine which types of data (e.g., processed sensor information versus raw measurements) should be transmitted under varying network conditions. A practical near-field ISAC roadmap should explicitly address coexistence between communication/ISAC waveforms and today’s mmWave radar operations, including mutual interference and harmonized spectrum-sharing strategies. New cross-layer mechanisms are required for user grouping, pilot allocation, and scheduling based on polar separability, as well as robust focusing under position uncertainty.
- **Mobility and dynamic environment considerations:** High-speed vehicular scenarios introduce rapid changes in channel conditions. Research should focus on mobility-aware operations, rapid adaptation to dynamic topologies, and the integration of ELAA sensing capabilities to predict and respond to environmental changes in real time. Multi-vehicle coordination and cooperative localization/sensing strategies remain largely unexplored in the near-field regime. Moreover, efficient algorithms are required to handle the dynamicity of vehicular scenarios with frequent variations, blockages, or partial occlusion of ELAAs.
- **Integration with network standards and protocols:** Practical deployment of ELAA-based ISAC requires harmonization with emerging 6G vehicular standards, such as NR-V2X- and 3GPP-defined sidelink procedures. Research should address protocol design, signaling overhead, and backward compatibility with existing vehicular communication infrastructure.

In summary, ELAA-enabled near-field ISAC offers significant potential to enhance communication, localization, and sensing in next-generation vehicular networks. The research directions outlined above highlight the multidimensional challenges, ranging from hardware implementation to signal processing, regulatory compliance, and system-level integration, that must be addressed to realize the full benefits of this technology.

## Figures and Tables

**Table 1:** Fraunhofer distance $r_{\mathrm{ff}}$ [m] for different array apertures and frequencies.

Aperture *D* [m]	5.9 GHz	10 GHz	28 GHz	60 GHz
1.5	89	150	420	900
2.0	157	267	747	1600
2.5	246	417	1167	2500

**Table 2:** Number of λ/2-spaced antenna elements required to cover different apertures *D*.

Aperture *D* [m]	5.9 GHz	10 GHz	28 GHz	60 GHz
1.5	59	100	280	600
2.0	79	133	373	800
2.5	98	167	467	1000

## Data Availability

No new data were created or analyzed in this study. Results are obtained from the analytical expressions provided in the paper. Data sharing is not applicable to this article.

## Abbreviations

The following abbreviations are used in this manuscript:

5G	fifth generation
6G	sixth generation
AI	artificial intelligence
AoA	angle of arrival
CAM	cooperative awareness message
CP	cyclic prefix
CPM	collective perception message
CRLB	Cramér–Rao lower bound
CSI	channel state information
DENM	decentralized environmental notification message
DoF	degrees of freedom
EIRP	equivalent isotropically radiated power
ELAA	extremely large aperture array
GNSS	global navigation satellite system
GPS	global positioning system
IMU	inertial measurement unit
ISAC	integrated sensing and communication
LOS	line-of-sight
LTE	long-term evolution
mmWave	millimeter-wave
MIMO	multiple-input multiple-output
MUSIC	multiple signal classification
NLOS	non-line-of-sight
NR	new radio
OFDM	orthogonal frequency-division multiplexing
RCS	radar cross section
RSU	road side unit
SNR	signal-to-noise ratio
SVD	singular value decomposition
TDoA	time-difference-of-arrival
ToA	time-of-arrival
TWR	two-way ranging
ULA	uniform linear array
V2V	vehicle-to-vehicle
V2X	vehicle-to-everything
VRU	vulnerable road user
XL-MIMO	extremely large-scale MIMO
